# Early responses given distinct tactics to infection of *Peronophythora litchii* in susceptible and resistant litchi cultivar

**DOI:** 10.1038/s41598-019-39100-w

**Published:** 2019-02-26

**Authors:** Jinhua Sun, Lulu Cao, Huanling Li, Guo Wang, Shujun Wang, Fang Li, Xiaoxiao Zou, Jiabao Wang

**Affiliations:** 1 Environment and Plant Protection Institute, Chinese Academy of Tropical Agricultural Sciences, Haikou, PR China; 2 Institute of Tropical Bioscience and Biotechnology, Chinese Academy of Tropical Agricultural Sciences, Haikou, PR China

## Abstract

Litchi downy blight, a destructive litchi disease caused by *Peronophythora litchii*, is controlled by intensive fungicide applying. Sources of resistance are used in conventional breeding approaches, but the mechanism is not well understood. Follow-up six years investigation, ‘Guiwei’ and ‘Heiye’ displayed stable susceptible and resistant against to *P*. *litchii*, respectively. After 72 hour inoculation, ‘Heiye’ showed few disease spots, while ‘Guiwei’ appeared brown and covered with white sporangia. Germination of sporangia and growth of mycelium in ‘Guiwei’ is more quickly than in ‘Heiye’. Transcript levels were measured at 6, 24, and 48 hour post-inoculation. ‘Oxidation-reduction process’ was dramatically enhanced in ‘Heiye’, which could promote its resistance to pathogen infection. A small ratio (3.78%) of common DEGs indicates that resistant and susceptible cultivars take different strategies to defense against *P*. *litchii*. At early infection stage, ‘Heiye’ induced a larger number of genes, including seven receptor-like kinases, which quickly recognized attack of pathogen and led to a rapidly resistance by regulation of degradation of proteasome, transcription factors, and cell wall remodeling. The early DGEs were exiguous in ‘Guiwei’, suggesting a weak response. Once the infection was successful, the resistance was repressed by down-regulated genes involved in phenylpropanoid metabolism, ET biosynthesis and signaling conduction in ‘Guiwei’. In conclusion, quickly recognition and early responses to pathogen, as well as minimal pathogen development and basal expression of resistance-related genes, were correlated with a high level of resistance in ‘Heiye’, while susceptible ‘Guiwei’ suffered massive infection due to lagging response and repressed signal transduction.

## Introduction

Litchi (*Litchi chinensis* Sonn.), a subtropical evergreen fruit tree of family Sapindaceae, originate from South China and has been widely cultivated in more than 20 countries due to delicious and nutritional value^1^. The planting area of litchi is approximately 0.59 million hectares in South China, of which yield annually 1.91 million tons fresh fruits. However, the fruits are highly susceptible to various diseases. Litchi downy blight, caused by *Peronophythora litchii*, is one of the major diseases in litchi^2^. *P*. *litchii* is an oomycete pathogen and exclusively infects litchi. The mycelium and oospore of *P*. *litchii* attacks fruits and results in watery brown spots^2,3^. The fungicides including carboxyl acid amide, mancozeb, cymoxanil, and metalaxyl have been extensively used to control litchi downy blight^4^. After a long time applying, the resistant isolates of *P*. *litchii* have been detected in some regions^5^. Considering both drug-resistance and environmental impact due to the fungicide applying, breeding of resistant cultivar to control the disease are required.

Detailed resistance mechanisms of plant have been described in a few model species. Plant responses to pathogens depend on recognition of microorganisms and induction of defense responses by downstream signal transduction. Pathogen invasion is firstly detected by the recognition of highly conserved pathogen-associated molecular patterns (PAMPs), leading to a basal resistance called PAMP-triggered immunity (PTI)^6^. The recognition of PAMPs is by cell membrane embedded pattern-recognition receptors (PRRs), which are either receptor-like kinases (RLKs) or receptor-like proteins (RLPs). PTI can be overcome by injecting type III effectors (T3Es) of pathogen into the host cells^7^. Recognition of T3Es is known as effector-triggered immunity (ETI)^7^. ETI can be accompanied by hypersensitive reaction (HR), a form of programmed cell death at the infection site that prevents pathogen spreading^8^. The HR thus triggers systemic acquired resistance (SAR) by salicylic acid (SA)-mediated defenses and confers broad-spectrum immunity to secondary infection^9^. Plant hormones are crucial systemic signals that strongly influence the level of plant resistance. SA, jasmonic acid (JA), and ethylene (ET) play vital roles in resistance to biotrophic and necrotrophic pathogens. The activation of hormone signaling will induce defense response, including stress responses, oxide-reduction processes, cell wall and wax biosynthesis processes, and pathogenesis-related proteins (PR)^10^. In addition, plant resistance is mediated by the proteolysis. The response of defense-related hormone, such as jasmonate, auxin, and abscisic acid signaling, are proteasome-dependent processes^11^. Receptor associated proteasomic degradation may be active in positive-regulation as well as negative-regulation^12^. Regulation of transcription factor involved in defense gene can also be regulated through the proteasome system^13,14^. Moreover, phytopathogenic pathogens can manipulate the proteasomal system for suppresses the PTI of host^15,16^.

At present, studies about litchi downy blight have mostly concentrated on biological characteristics of pathogen, chemical control and screening of resistant cultivars in litchi^17,18^. Little is known about the disease resistance-relevant genes involved in the interaction between host and pathogen, which requires exploration into the resistance mechanisms against *P*. *litchii*. In this paper, the disease process of litchi downy blight was firstly investigated in two litchi cultivars (‘Heiye’ and ‘Guiwei’) by Scanning Electron Microscopy (SEM). And then, transcriptome sequencing was performed via *de novo* RNA-seq technology at three stages (6, 24, and 48 hour post inoculation). Differential gene expression was conducted to identify the disease resistance-relevant genes and metabolic pathways involved in *P*. *litchii* infection. These genes and pathways will provide a theoretical basis for expounding the resistance mechanism against litchi downy blight in litchi.

## Results

### Determination of the resistance of litchi cultivars

**Figure 1:**
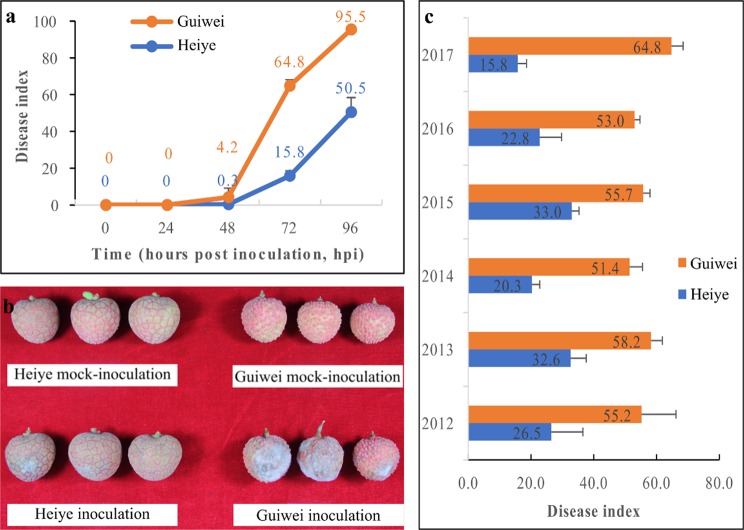
Diseasse symptom and disease index between two cultivars. (**a**) Disease index increase after inoculation with *P*. *litchii* in 2017, (**b**) Disease symptom of two cultivars inoculated or mocked-inoculated at 72 hpi, (**c**) Disease index of two cultiavrs across six years.

After inoculated with *P*. *litchii*, the disease severity of two cultivars was assessed according the disease index (DI) in the greenhouse (Fig. 1). A few white spots both appeared in the exocarp of two cultivars at 48 hour post inoculation (hpi) (Fig. 1a). Since then, the progress of disease accelerated. The disease severity was significantly different between two cultivars at 72 and 96 hpi (Fig. 1a). Disease resistance of two cultivars was determined by the DI at 72 hpi. The infected fruits of ‘Heiye’ showed small spots, while ‘Guiwei’ covered with masses of downy white sporangia and sporangiophores (Fig. 1b). Moreover, six years follow-up survey displayed that the DI of ‘Heiye’ were 15.8–33.0 and the DI of ‘Guiwei’ were 51.4–64.8 (Fig. 1c). The results indicate that the ‘Heiye’ is a resistant cultivar, whereas ‘Guiwei’ is a susceptible cultivar.

### Microscope observation on pathogen infection

**Figure 2:**
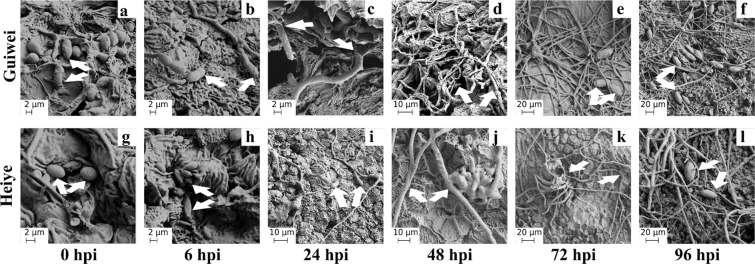
Microscope observation of infection of *P*. *litchii* between two cultivars at six time-points. (**a**,**g**,**h**) The inoculated spores, (**b**,**i**) the germinated sporangia, (**c**,**d**,**j**,**k**) the mycelium, (**e**,**f**,**l**) the new sporangia.

The infection process of *P*. *litchii* in two cultivars with inoculation was monitored by SEM method. The spores were both observed in the exocarp of two cultivars after inoculation with *P*. *litchii* (Fig. 2a,g). At the 6 hpi, germination of sporangia was observed in ‘Guiwei’ but not in ‘Heiye’ (Fig. 2b,h). At the 24 hpi, the sporangia extend out of the mycelium and grow along cracked lobe valley in ‘Guiwei’, while the sporangia just started to germinate in ‘Heiye’ (Fig. 2c,i). At the 48 hpi, a lot of mycelium were formed in ‘Guiwei’, while the same thing was observed in ‘Heiye’ until 72 hpi, and a few mycelia grow along the surface of exocarps in ‘Heiye’ (Fig. 2d,j,k). At the 72 hpi, the mycelium reproduced a few sporangia in the exocarp of ‘Guiwei’ (Fig. 2e). At the 96 hpi, much more sporangia were formed in ‘Guiwei’, while only a few reproduced sporangia were observed in ‘Heiye’ (Fig. 2f,l). Microscopic observations revealed that cortical infection was both present in ‘Heiye’ and ‘Guiwei’ at 6, 24 and 48 hpi (Fig. 2). For this reason, these time-points (6, 24, and 48 hpi) were selected to investigate the differential transcript changes among cultivars and inoculation.

### RNA Sequencing and *De novo* Assembly

Approximately 11.93–17.35 million 125-bp paired-end reads were generated from the 36 samples through RNA sequencing (Table S2). The GC content of the sequence data from the 36 libraries ranged from 46.20 to 50.50%, and the Q30 values (reads with an average quality scores >30) were all ∼90%, indicating that the quality and accuracy of sequencing data was sufficient for further analysis. After sequence trimming and redundance removed, the retained high-quality reads of all samples were *de novo* assembled into 32614 unigenes as reference transcripts. The N50 of the assembled genes was 1466 bp, with an average length of 1110 bp and a maximum length of 11441 bp (Table 1). The percentage of sequenced reads from all libraries remapped to the assembled reference transcripts was ∼70% (Table S2). The 30006 of unigenes were functionally annotated in at least one database with an e-value cutoff of 1e^−5^ (Table 1, Table S3). The sequencing data has been deposited into NCBI sequence read archive (SRA) under BioProject accession PRJNA450886.

**Table 1:** Summary of transcriptome assembly for litchi.

Category	Count
Clean read pairs (million)	531.51
Unigenes	32,614
Maximum length of unigene (bp)	11,441
Minimum length of unigene (bp)	201
Mean length of unigene (bp)	1,110
Unigenes size N50 (bp)	1,466
GC content of genes (%)	42.85

### Inter-genotypes differences in basal gene expression

On the plot of Principal Component Analysis (PCA), shorter distance between two points indicates greater similarity of gene expression profiles between two samples. PCA showed that all samples of ‘Heiye’ clearly stratify away from those of ‘Guiwei’ in the first principal component, indicating large differences in the gene transcription patterns of these two cultivars. The second principal components show a further separation between 6 hpi and the others (24 and 48 hpi) in ‘Guiwei’, in which the inoculation showed no-significant changes from mock-inoculation (Fig. S1).

**Figure 3:**
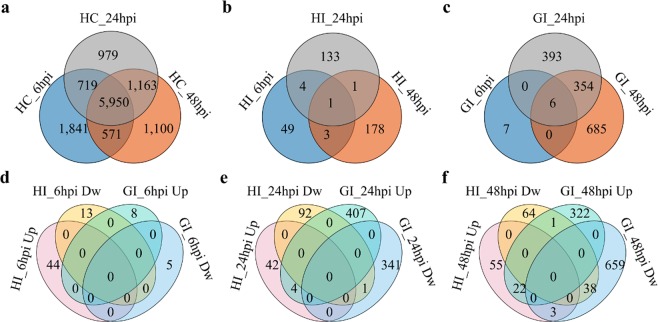
Venn diagrams showing the overlap of the differentially expressed genes (DEGs; fold change; | FC | ≥ 2). (**a**) The DEGs (HC; HC_vs_GC) between ‘Heiye’ and ‘Guiwei’ with mock-inoculation; (**b**) the DEGs (HI; HI_vs_HC) between inoculation with *P*. *litchii* and mock-inoculation in ‘Heiye’; (**c**) the DEGs (GI; GI_vs_GC) between inoculation with *P*. *litchii* and mock-inoculation in ‘Guiwei’; (**d**,**f**) the DEGs were up-regulated (Up) and down-regulated (Dw) at 6, 24, and 48 hpi in ‘Heiye’ (HI) and ‘Guiwei’ (GI) inoculated with *P*. *litchii*.

The uninoculated ‘Heiye’ and ‘Guiwei’ were further compared to analyze the basal gene expression pattern (Figs 3a, S2). Three pairwise comparisons of different inoculation times (HC_vs_GC_6hpi, HC_vs_GC_24hpi, and HC_vs_GC_48hpi) were generated to identify differential expression genes (DEGs) between two cultivars. Differential expression analysis revealed a total of 9081, 8811, 8784 DEGs identified respectively at 6, 24, and 48 hpi (Figs 3a, S2). The total numbers of DEGs was decreased slightly with time past. There were 12323 non-redundant DEGs and 5950 common DEGs in three time-points (Figs 3a, S2). Moreover, among all DEGs, 3997 unigenes had a much higher expression value in resistant cultivar, and a total of 1925 unigenes had a much higher expression value in susceptible cultivar (Fig. S2).

As inter-genotype differences may reflect the mechanisms of disease resistance and susceptibility, we also classified these DEGs according to functional categories. Following the Nr annotations, the DEGs were mapped into three Gene ontology (GO)^19^ categories, of which fifteen sub-categories were significantly enriched (corrected P-value ≤ 0.01) in biological process (Table S4). DEGs involved in ‘Oxidation-reduction process’ made up the largest groups, and the up-regulated DEGs were also enriched in this GO term (Table S4). The ‘Defense response’, ‘Response to stress’, and ‘Chitin metabolic process’ were both enriched in total and down-regulated DEGs analysis (Table S4).

Moreover, Kyoto Encyclopedia of Genes and Genomes (KEGG)^20^ analyses were performed to identify the basal level biological pathways in litchi pericarp. The 1145 out of 5922 DEGs had KEGG Orthology (KO) IDs and could be categorized into 133 pathways (data not show). A total of 7 pathways were significantly enriched (corrected P-value ≤ 0.05). Genes involved in ‘Plant-pathogen interaction’, ‘alpha-Linolenic acid metabolism’, ‘Cysteine and methionine metabolism’, ‘Brassinosteroid biosynthesis’, ‘Indole alkaloid biosynthesis’, ‘Anthocyanin biosynthesis’, and ‘Tyrosine metabolism’ were the most represented DEGs (Supplementary Table S5).

**Figure 4:**
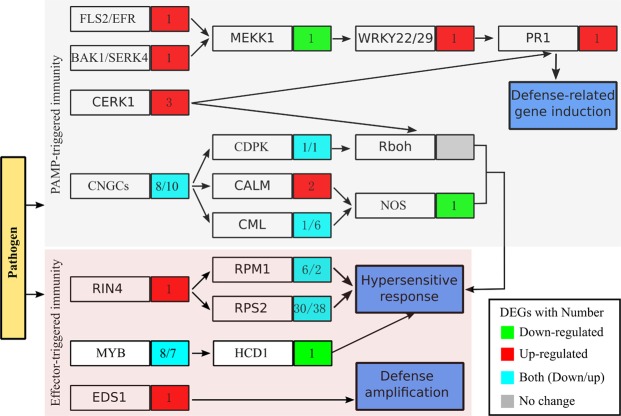
The diagram of the ‘Plant-pathogen interaction’ between ‘Heiye’ and ‘Guiwei’. The color rectangles represent trend of genes, in which the number indicate the amount of differentiated gene. Green represent down-regulated in ‘Heiye’, red represent up-regulated in ‘Heiye’, blue represent that both down- and up-regulated members of gene family were found in ‘Heiye’, gray represent no DEG between two cultivars.

In the present study, ‘plant-pathogen interaction’ pathway was significantly enriched and exhibited the most significantly different expression levels between the resistant and susceptible cultivars. 140 DEGs were found in ‘plant-pathogen interaction’ pathway, of which 80 and 60 were respectively up- and down-regulated (Fig. 4, Tables S5–6. Among them, sixty-eight disease resistance protein RPS2 (Resistance to *Pseudomonas*. *syringae* 2) made up the largest group (Fig. 4, Table S6). Another eight disease resistance proteins RPM1 (Resistance to *Pseudomonas syringae pv maculicola* 1) were also differential expression between two cultivars (Fig. 4, Table S6). One *RIN4* (RPM1-interacting protein 4) associating with both *RPS2* and *RPM1* was up-regulated. One *PR1* was also up-regulated (Fig. 4, Table S6). The second large group was *CNGC* (cyclic nucleotide gated channel) including eighteen members, and fifteen transcription factor *MYB* were made up the third large groups. The number of up- and down-regulated genes were comparable in these two groups (Fig. 4, Table S6). Three sub-families of Ca^2+^ sensors including *CALM* (calmodulins), *CDPK* (calcium dependent protein kinases) and *CML* (calcium-binding protein) were identified (Fig. 4, Table S6). Two *CALM*s, one *CDPK*, and six of seven *CML*s were up-regulated, while only one *CDPK* and one *CML*s were down-regulated. One *MEKK1* (mitogen-activated protein kinase kinase kinase 1), one *NOS1* (nitric-oxide synthase), and one *HCD1* (very-long-chain (3R)-3-hydroxyacyl-CoA dehydratase) were down-regulated. the rest six groups, which include four *CTSF* (cathepsin), three *CERK1* (chitin elicitor receptor kinase 1), one *WRKY22*, one *FLS2* (flagellin-sensitive 2), one *EDS1* (enhanced disease susceptibility 1 protein), and one *BAK1* (brassinosteroid insensitive 1-associated receptor kinase 1), were just highly expressed in ‘Heiye’ (Fig. 4, Table S6).

### Differentially Expressed Genes after inoculation

**Figure 5:**
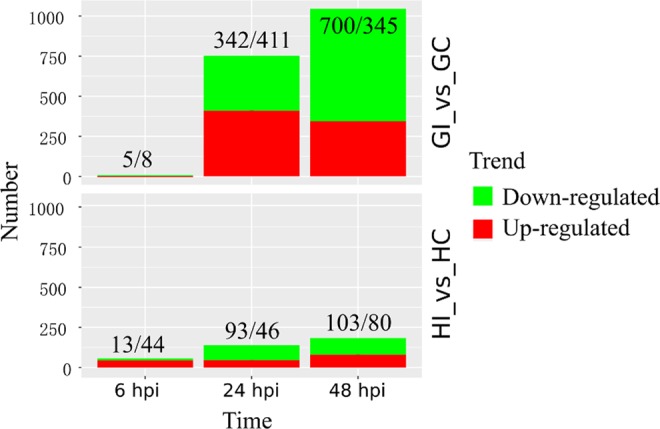
The number of DEGs comparison inoculation with mock-inoculation in two cultivars at three time-points. Green represent down-regulated after inoculation, red represent up-regulated after inoculation. The upper number indicate the amount of down-regulated DEGs, the lower number indicate the amount of up-regulated DEGs.

To find genes that were induced by *P*. *litchii*, pairwise comparisons were made between inoculation with *P*. *litchii* and mock-inoculation at the same time-point in each cultivar. A total of 1717 DEGs were identified, of which 1445 and 369 were regulated in ‘Guiwei’ and ‘Heiye’ respectively (Figs 3b,c and 5). Notably, when all DEGs of ‘Heiye’ were compared to those of ‘Guiwei’, only 97 unigenes were both regulated in two cultivars (Fig. 3d,e,f). No common DEGs was identified in two cultivars at 6 hpi (Fig. 3d). Five common DEGs were identified at 24 hpi, of which four were both up-regulated, and one was down-regulated (Fig. 3e). Sixty-four common DEGs were identified at 48 hpi, of which twenty-two were up-regulated, and thirty-eight were down-regulated, and three were up-regulated in ‘Heiye’ but down-regulated in ‘Guiwei’, and one was down-regulated in ‘Heiye’ but up-regulated in ‘Guiwei’ (Fig. 3f). At 24 hpi, there were four DEGs (c36949_c0_g1, c8306_c0_g2, c36893_c0_g1, and c13847_c0_g1) up-regulated in two cultivars. The c36949_c0_g1 and c8306_c0_g2 were encoded EXL2 (EXORDIUM-like 2 protein). The c36893_c0_g1 was encoded an AOC (allene oxide cyclase). The function of c13847_c0_g1 was unknown. One gene (c5397_c0_g3) was down-regulated in two cultivars at 24 hpi, which function was unknown (Table S7).

### DEGs response to *P*. *litchii* in susceptible cultivar

Thirteen (0.90%), 753 (52.11%), and 1045 (72.32%) DEGs were identified in ‘Guiwei’ at 6, 24 and 48 hpi, respectively. Only 8 (61.54%), 411 (54.58%), and 345 (33.01%) were up-regulated (Figs 3c and 5). There were six DEGs induced across three time-points, of which one (c20888_c0_g1) encoded (+)-neomenthol dehydrogenase (SDR1), one (c3313_c0_g1) encoded LRR receptor-like serine/threonine-protein kinase (LRR-RLK, GSO2), two (c22648_c0_g1 and c22646_c0_g1) encoded galactinol synthase 2 (GolS2), one (c44404_c0_g1) encoded galactinol sucrose galactosyltransferase 5, and one (c3790_c0_g1) encoded stachyose synthase (Figs 3c, S3). The two of former were up-regulated at 6 hpi, and down-regulated at 24 hpi, and up-regulated at 48 hpi (Fig. S3). The four of latter were down-regulated at 6 and 48 hpi, and up-regulated at 24 hpi (Fig. S3).

**Figure 6:**
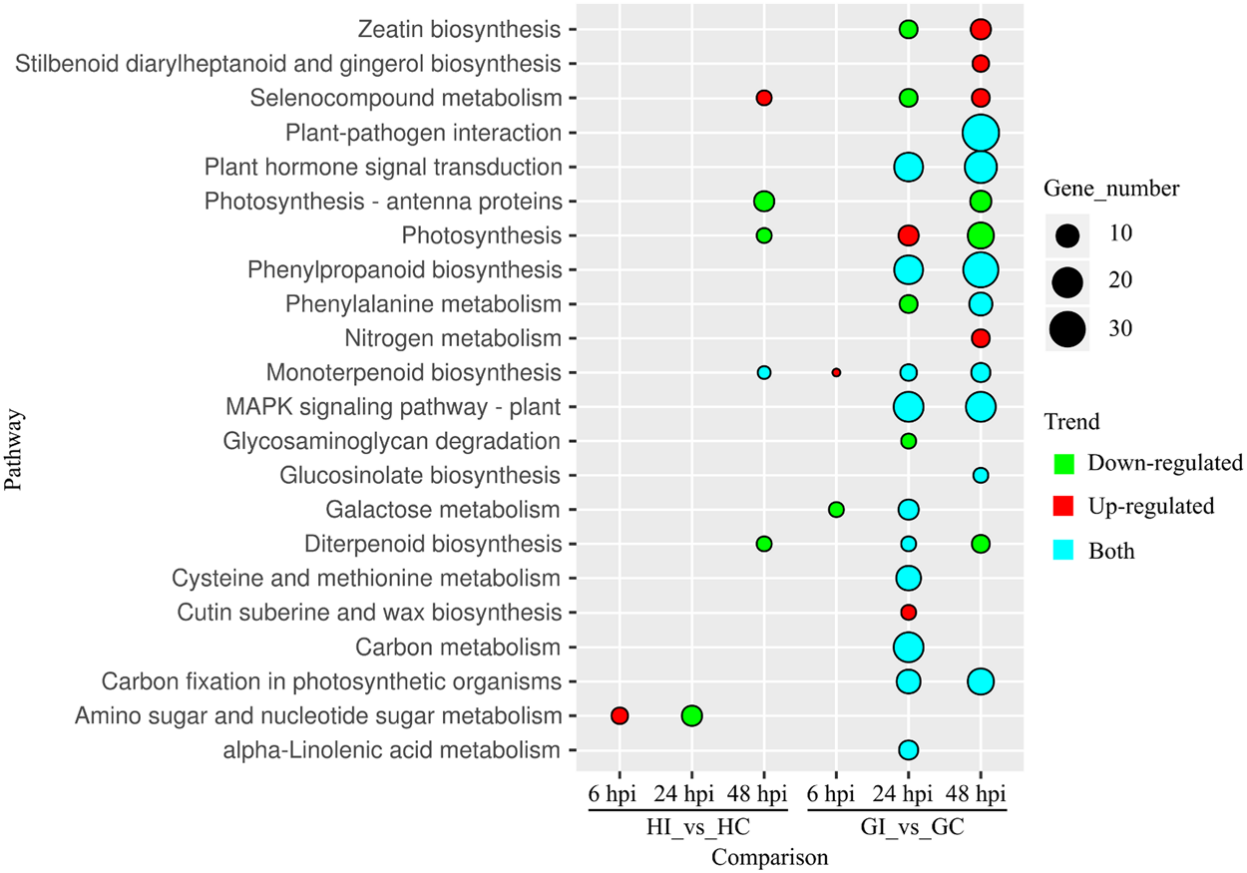
The significantly enriched KEGG pathways of DEGs respond to *P*. *litchii* infection in two cultivars. Area of circle represent the number of DEGs in related pathways. Green represent down-regulated, red represent up-regulated, blue represent that both down- and up-regulated members of pathway were found after inoculation.

KEEG enrichment show that twenty-one pathways were significant difference after inoculation with *P*. *litchii* in ‘Guiwei’ (Fig. 6, Table S8). The ‘Galactose metabolism’ (ko00052) was down-regulated enriched at 6 hpi but up-regulated at 24 hpi (Fig. 6, Table S8). The major DEGs involved in ‘MAPK signaling pathway - plant’ (ko04014) and ‘Plant hormone signal transduction’ (ko04075) were down-regulated consistently at 24 and 48 hpi (Fig. 6, Tables S8, S9, S10). The ‘Monoterpenoid biosynthesis’ (ko00902) was up-regulated enriched at 6 and 24 hpi but down-regulated at 48 hpi (Fig. 6, Table S8). The major DEGs involved in ‘Photosynthesis’ (ko00195), ‘Carbon fixation in photosynthetic organisms’ (ko00710), and ‘Diterpenoid biosynthesis’ (ko00904) were up-regulated at 24 hpi but down-regulated 48 hpi (Fig. 6, Table S8). While ‘Phenylalanine metabolism’ (ko00360), ‘Selenocompound metabolism’ (ko00450), ‘Zeatin biosynthesis’ (ko00908), and ‘Phenylpropanoid biosynthesis’ were down-regulated at 24 hpi but up-regulated at 48 hpi (Fig. 6, Table S8). The major DGEs involved in ‘Cysteine and methionine metabolism’ (ko00270) and ‘alpha-Linolenic acid metabolism’ (ko00592) were down-regulated at 24 hpi (Fig. 6, Table S8). Additionally, nineteen of thirty-four DEGs involved ‘Plant-pathogen interaction’ (ko04626) were also down-regulated at 48 hpi (Fig. 6, Table S8).

The ‘MAPK signaling pathway - plant’ was the most enrichment pathway response to *P*. *litchii* in ‘Guiwei’ at 24 hpi (Figs 6, S4, Table S8). Twenty-one DEGs were identified in this pathway at 24 hpi, of which 16 and 5 were down- and up-regulated respectively (Fig. 6, Table S8), whereas twenty-one DEGs in 48 hpi, while 11 and 10 were respectively down- and up-regulated (Fig. 6, Table S8). Among them, one *NDK* (nucleoside-diphosphate kinase) and five *PP2C*s (protein phosphatase 2C) were up-regulated, while the rest of fifteen DEGs, three *FLS2*s, two *RBOH*s (respiratory burst oxidase), one *PR1*, one *PYL* (abscisic acid receptor), three *ETR*s (ethylene receptor), three *EBF1_2*s (EIN3-binding factor), and two *ERF1*s (ethylene-responsive transcription factor 1), were down-regulated (Figs S4–6, Table S9). The ‘Plant hormone signal transduction’ and ‘Phenylpropanoid biosynthesis’ both contained nineteen DEGs and enriched significantly (Figs S6–8, Tables S8, 10–11). In ‘Plant hormone signal transduction’, except the DEGs mentioned in ‘MAPK signaling pathway-plant’, one *ARF* (auxin response factor), one *TCH4* (xyloglucan:xyloglucosyl transferase), and one *NPR1* (non-expressor of pathogenesis-related genes 1) were up-regulated and one *BSK* (BR-signaling kinase) was down-regulated (Fig. S6, Table S10). In ‘Phenylproanoid biosynthesis’ pathway, except one *COMT* (caffeic acid 3-O-methyltransferase) and one *POD* (peroxidase) were up-regulated, the rest of seventeen DEGs, such as three *PAL*s (phenylalanine ammonia-lyase), one *HST* (shikimate O-hydroxycinnamoyltransferase), one *CCoAOMT* (caffeoyl-CoA O-methyltransferase), one *CSE* (caffeoylshikimate esterase), and one *4CL* (4-coumarate–CoA ligase), were down-regulated at 24 hpi (Figs S7–8, Table S11). Interestingly, the profile of phenylpropanoid metabolism, ET related genes, and *PP2C*s at 24 hpi was reversed at 48 hpi (Tables S6–7).

### DEGs response to *P*. *litchii* in resistant cultivar

‘Heiye’ inoculated with *P*. *litchii* showed 57 (15.45%), 139 (37.67%), and 183 (49.59%) DEGs at 6, 24 and 48 hpi, respectively; among them 44 (77.19%), 46 (33.09%), and 80 (43.72%) were up-regulated (Figs 3b and 5). The results showed that the remarkable changes in the transcriptome profile occurred at 6 hpi in ‘Heiye’. The number of DEGs at 6 hpi in ‘Heiye’ was markedly more than those in other two time-points. One DEGs (c13031_c0_g1) encoding a receptor-like kinase *EP1* with mannose-binding domain was regulated at three time-points, which was up-regulated (FC = 2.28) at 6 hpi, then down-regulated (FC = −1.58) at 24 hpi, and up-regulated (FC = 1.60) at 48 hpi (Fig. S3).

As a KEEG enrichment of DEGs response to *P*. *litchii* in ‘Heiye’, only six pathways showed significant difference (corrected P-value ≤ 0.01) (Fig. 6, Table S8). The up-regulated DEG was enriched in ‘Amino sugar and nucleotide sugar metabolism’ pathway at 6 hpi, and the down-regulated DEGs in this pathway enriched at 24 hpi (Table S8). In fact, the chitinases (all five at 6 hpi and seven out of eight at 24 hpi) made up the most of DEGs in ‘Amino sugar and nucleotide sugar metabolism’ pathway. At the 48 hpi, ‘Photosynthesis’ and ‘Diterpenoid biosynthesis’ were down-regulated, and ‘Selenocompound metabolism’ was up-regulated (Fig. 6, Table S8).

**Figure 7:**
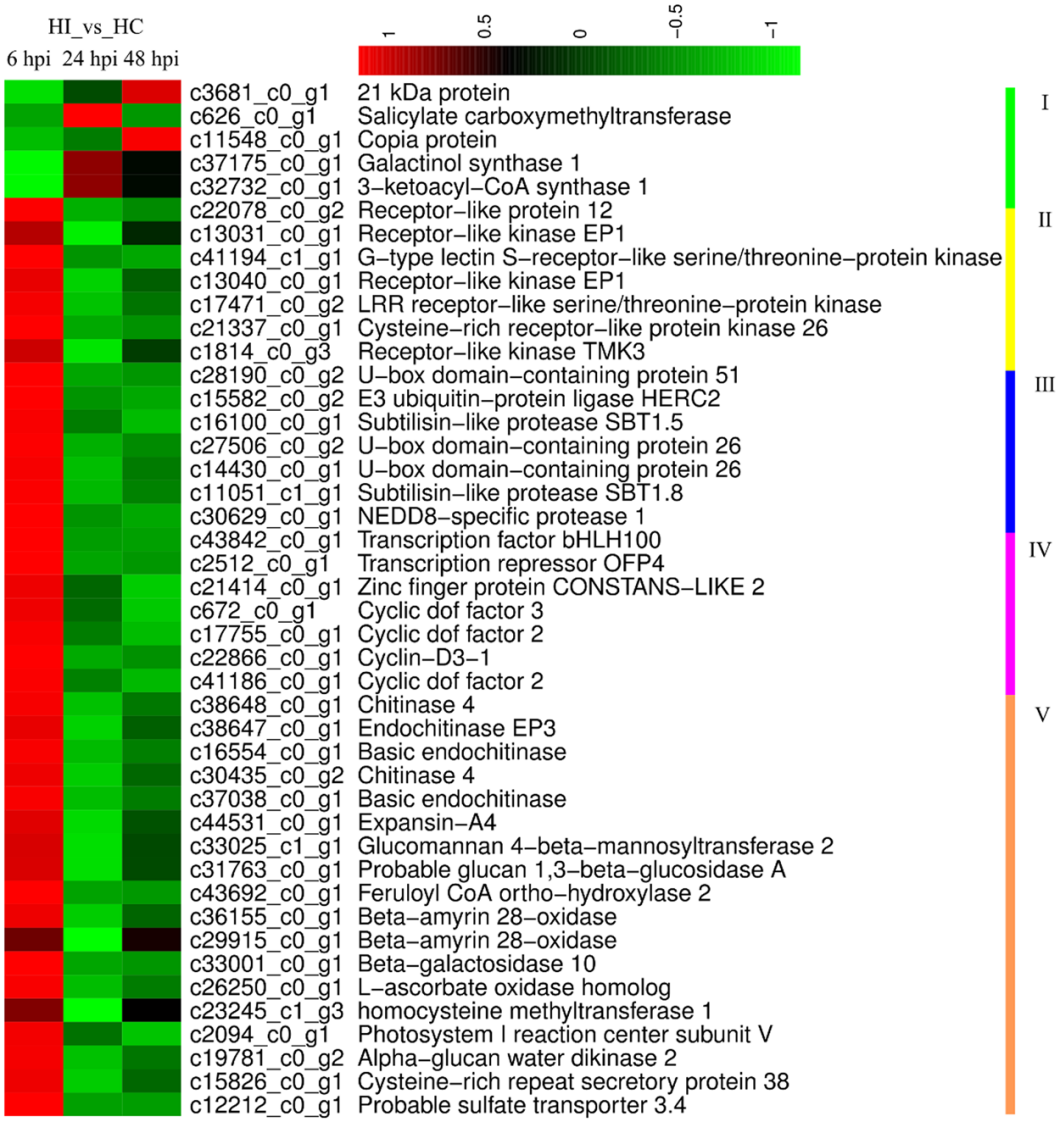
The DEGs respond to infection of *P*. *litchii* in ‘Heiye’ at early stage. The forty-four DEGs with annotation divide into five groups. I, down-regulated DEGs, II, receptor-like kinases, III, proteases, IV, transcription factors, V, other up-regulated DEGs.

To obtain an overview of the process respond to infection of *P*. *litchii* during early stages, the DEGs were further analysis in ‘Heiye’ at 6 hpi. According profile of expression and function, the 44 of 57 DEGs with annotation were classified into five groups (Fig. 7). Except the group 1 was down-regulated, the other groups were all up-regulated. The group 1 were made up with five DEGs including a *GolS* (c32732_c0_g1), *SAMT* (salicylate carboxymethyltransferase, c626_c0_g1), and 3-ketoacyl-CoA synthase 1 (*KCS1*, c32732_c0_g1). The group 2 were consisted with seven *RLK*s (c1814_c0_g3, c22078_c0_g2, c21337_c0_g1, c17471_c0_g2, c13031_c0_g1, c13040_c0_g1, and c41194_c0_g1). The group 3 were consisted with seven protease proteins, including one E3 ubiquitin-protein ligase (c15582_c0_g2), two U-box domain-containing protein (c28190_c0_g2, c27506_c0_g2, and c14430_c0_g1), two Subtilisin-like protease (*SBT*s, c16100_c0_g1 and c11051_c1_g1), and one *NEDD8*-specific protease (c30629_c0_g1). The group 4 were transcription factor including three cyclic dof factor (c17755_c0_g1, c41186_c0_g1, and c672_c0_g1), one *bHLH100* (c43842_c0_g1), and one transcription repressor *OFP4* (c2512_c0_g1), and one zinc finger protein CONSTANS-LIKE 2 (c21414_c0_g1). The group 5 were the rest such as five chitinases (c38647_c0_g1, c16554_c0_g1, c37038_c0_g1, c30435_c0_g2, and c38648_c0_g1), and one cysteine-rich repeat secretory protein 38 (c15826_c0_g1), expansin-A4 (c44531_c0_g1), Glucomannan 4-beta-mannosyltransferase 2 (*CSLA2*, c33025_c1_g1), Glucan 1,3-beta-glucosidase (c31763_c0_g1), Feruloyl CoA ortho-hydroxylase 2 (*F6’H2*, c43692_c0_g1), L-ascorbate oxidase (c26250_c0_g1), and galactosidase (c33001_c0_g1) etc.

### Validation of transcripts by real-time PCR

To validate the expression profiling by RNA-seq, the expression levels of six genes, including two peroxidase genes, one phenylalanine ammonia-lyase gene, one beta-glucosidase gene, one caffeoyl-CoA O-methyltransferase gene, and one feruloyl-CoA ortho-hydroxylase gene, were further analysed by qRT-PCR (Fig. S9, Table S12). All the genes showed differential expression levels between ‘Guiwei’ and ‘Heiye’. Correlation analysis was performed between FPKM by RNA-seq and relative expression by qRT-PCR for each gene. The Pearson’s correlation coefficient between the data generated from the two platforms was high (R2 value in range of 0.75 to 0.97), indicating that the RNA-seq approach provided reliable differential gene expression information (Fig. S9).

## Discussion

In the present study, the distinct colonization of *P*. *litchii* on litchi pericarps was first revealed by SEM over a time series. In susceptible cultivar, *P*. *litchii* was directly penetrated the exocarps and established a primary restricted infection before 6 hpi (contact phase and penetration phase). Subsequently, the pathogen initiated a massive outgrowth before 72 hpi (incubation phase) and formed a lot of sporangium until 96 hpi (symptom appearance phase). Conversely, penetration of *P*. *litchii* on resistant cultivar was not detected at 6 hpi (contact phase) and showed a substantial delay until 24 hpi (penetration phase). This lagging of infection resulted in markedly lower germination and infection rates in resistant cultivar than susceptible cultivar at the following stages. Additionally, the sporangium was also formed but much smaller in resistant than susceptible cultivar at 96 hpi (symptom appearance phase). The resistant cultivar is characterized as a long contact phase and a short incubation phase (24–72 hpi) with a less DI. It indicates that the germination of sporangia and the growth of mycelium in susceptible cultivar is more quickly than in resistant cultivar. It should be noted that the inoculation pore suspension (1 × 10^4^ spores·mL^−1^) of *P*. *litchii* were higher content than the spores in the fields. The high spore content can accelerate the progress of disease under lab condition. In fact, the downy blight was less happened in resistant cultivar ‘Heiye’ than in susceptible cultivar ‘Guiwei’ in fields investigation (data not showed). Therefore, it seems that the disease is obstructed through blocked colonization of *P*. *litchii* on resistant cultivar during early infection stages.

There was a small ratio (3.78%, 65/1717) of common DEGs respond to infection of *P*. *litchii* between two cultivars. Especially, there is no common DEGs at early infection stage (6 hpi). It indicates that resistant and susceptible cultivar take different strategies to defense against *P*. *litchii* at contact phase. At 24 hpi, the infection was both complete in ‘Guiwei’ and ‘Heiye’. In this study, two *EXL2* and one *AOC* were both up-regulated response to *P*. *litchii* attack in two cultivars at 24 hpi. *EXL2* play a role in a brassinosteroid (BR)-dependent regulation. BR can stimulate photosynthesis and cell expansion in plant. One *EXL2* is highly expression and involved in cell wall remodeling in litchi^21^. In *Arabidopsis*, *EXL1*, homolog of *EXL2*, is involved in a primary response to energy deprivation and reduced BR-dependent growth^22^. *AOC* is involved in the production of 12-oxo-phytodienoic acid, a precursor of jasmonic acid. It suggests that the BR and JA take part in response to infection of *P*. *litchii* in litchi. There were sixty common DEGs between two cultivars at post-incubation phase (48 hpi). Four methyltransferases and two dehydratases were up-regulated, while seventeen genes related to chloroplast were down-regulated. It suggests that the cell wall of litchi was degrade by activated methyltransferase activity and photo reaction were repress at post-incubation phase.

After establishing that ‘Heiye’ could effectively block *P*. *litchii* and that ‘Guiwei’ was a favorable host, the underlying possible mechanisms of resistance in ‘Heiye’ and susceptibility in ‘Guiwei’ were investigated. PCA analysis indicated that the major difference between two cultivars attributed to genotype variation. Further analysis showed that the ‘plant-pathogen interaction’ pathway was significantly enriched as an inter-genotype difference. Plant defense responses are divided into two categories: PTI and ETI^7^. PTI is triggered by the recognition of generally conserved elicitors such as chitin and flagellins^23^. *FLS2* is an *RLK* recognized flagellin of bacterial and acts as a key regulator of PTI triggered by diverse PAMPs. *BAK1*, a ligand-independent coreceptor of *RLK*, and *CERK1* are also a central regulator of PTI. In agreement with this condition, One *FLS2*, one *BAK1*, and three *CERK1* genes were higher expression in resistant cultivar than in susceptible cultivar. The downstream signaling activates one *WRKY22*, which driving highly expression of *PR1*. Furthermore, eight genes involved in Ca^2+^ signaling pathway, which of two *CALM*s and six of seven *CML*s, were up-regulated in ‘Heiye’. It suggests that high *FLS2* activity together with *BAK1*, *CERK1*, *WRKY22* and Ca^2+^ signaling may contribute to PTI in basal defense in resistant cultivar.

Host resistance can regulate through the reactive oxygen species (ROS). *CERK1*, as well as *GNGC*s, could activated ROS signaling pathway. In our study, eighteen of *CNGC*s were differentially expressed between two cultivars. The ‘Oxidation-reduction process’ was dramatically enhanced in ‘Heiye’, which was consistent with the responses of wheat resistance against *Heterodera avenae*^24^. It seems that the reliably high level of ROS produced by basal gene expression in ‘Heiye’ could, at least in part, promote its resistance to pathogen infection, while the weak ROS in ‘Guiwei’ may contribute to its susceptibility. On other hand, *CNGC*s and ETI induced the hypersensitive response to block the pathogen attack. A large of ETI related genes, such as *RIN4*, *MYB*, *EDS1*, *RPM1*, and *RPS2*, were differential expressed between two cultivars. It’s important to note that one *EDS1* was highly expressing in ‘Heiye’. *EDS1* acts redundantly with salicylic acid and positive regulate both basal resistance and effector-triggered immunity^25,26^. The differential expression of ETI-related genes may also contribute to basal defense in resistant cultivar.

The pathogen infected successfully exocarp of ‘Guiwei’ at 6 hpi. At this time, only a few DEGs responding to infection of *P*. *litchii* were detected, indicating that response to infection of *P*. *litchii* is feeble in susceptible cultivar. Six DEGs expressed differential at three point-times. Among them, one *SDR1* encoding (+)-neomenthol dehydrogenase and one *GSO2* encoding LRR-RLK were up-regulated. *SDR1* not only catalyze a menthone reduction to produce neomenthol but also possess activity for neomenthol oxidation. *AtSDR1* positively regulate defenses against a broad spectrum of pathogens in *Arabidopsis*^27^. *GSO2* can positively regulate cell proliferation by intercellular signaling^28^. Moreover, as an LRR-RLKs, *GSO2* may have a function to recognize attack of pathogen. These two genes reflect feedback to infection of *P*. *litchii* in susceptible cultivar. Whereas another four genes, which were involved in synthesis of raffinose family of oligosaccharides (RFOs), were all down-regulated. RFOs are currently emerging as crucial molecules during stress response in plants. Biosynthesis of RFOs begin with the activity of galactinol synthase (GolS). The *GolS1*-overexpressing transgenic tobacco plants enhanced resistance against the pathogens *Botrytis cinerea* and *Erwinia carotovora*^29^. Further study demonstrated that galactinol and RFOs act as signaling component for induced systemic resistance against *B*. *cinerea* infection in *Arabidopsis*^30^. It suggests that the successful infection of *P*. *litchii* disturb the resistant action by down-regulated RFOs related genes at penetration phase in susceptible cultivar.

At 24 hpi, most genes related to ET biosynthesis and signaling conduction were suppressed in ‘Guiwei’. ET plays diverse roles in plant defense response^9^. One *SAM* and two *ACO* genes, which involved in the ethylene biosynthesis, were strongly down-regulated in ‘Guiwei’ after infection of *P*. *litchii*. Not only the synthesis of ET was inhibited but also the ethylene signaling was repressed. Three *ETR*s were down-regulated. ETRs percept the ET and act as regulator of ethylene signaling. ERFs are the major downstream regulatory factors of ET signaling pathway in stress-responses. The expression of *ERF1* was induced by the transcription factor *EIN3* (ETHYLENE INSENSITIVE3)^31^. EIN3-binding F-box protein (*EBF1_2*) may mediate E3 ubiquitin ligase complexes and subsequent degradation of target proteins. Three *EBF1_2*s and two *ERF1* genes were down-regulated upon *P*. *litchii* attack at 24 hpi. Moreover, one *PR-1* gene mediated by *ERF1* was negatively-regulated. On the other hand, phenylpropanoid metabolism, which is central to secondary metabolite production of defense-related compounds, was inhibited. The phenylpropanoid genes in key lignin formation, such as *PAL*, *HST*, *CCoAOMT*, *CSE*, *4CL*, *POD*, were mostly repressed in susceptible cultivar at 24 hpi. This change not only reduced the defense-related compounds but also effected the content of related hormones. In plants such as poplar, SA synthesis mainly takes place through phenylalanine ammonia-lyase (*PAL*)-dependent phenylpropanoid pathway^32^. In our study, three *PAL*-encoding genes were down-regulated in ‘Guiwei’ at 24 hpi. It suggests that infection of *P*. *litchii* repress the resistant action by down-regulated phenylpropanoid metabolism related genes, ET-related biosynthesis and signaling conduction at early stage of incubation phase in susceptible cultivar.

ABA is known to stimulate responses to several abiotic stresses (drought, salt and cold) as well as seed germination and plant growth^33^. The application of exogeneous ABA increases the susceptibility to pathogens. Additionally, ABA-deficient mutants showed a reduction in susceptibility to *Hyaloperonospora parasitica* in *Arabidopsis*^34^. Collectively, ABA behaves as a negative regulator of defence responses. ABA binding to PYR/PYL/RCAR intracellular receptors leads to inhibition of *PP2C*s, causing the activation of the ABA signaling pathway. There were one down-regulated *PYL* and five up-regulated *PP2C*s in ‘Guiwei’ after inoculation, indicating that the ABA mediated susceptibility was repressed. ABA deficiency activates *NPR1*-dependent resistance to *Psm ES4326* in *Arabidopsis*^35^. The transcription coactivator *NPR1* is a master regulator of basal and systemic acquired resistance in plants. Overexpression of *NPR1* in *Brassica juncea* enhanced resistance to *Alternaria brassicae* and *Erysiphe cruciferarum*^36^. One *NPR1* were up-regulated in ‘Guiwei’ after infection of *P*. *litchii*. *NPR1* regulates positively the majority of salicylic acid (SA)-dependent signaling pathway and negatively regulates JA-dependent signaling pathway. It suggests that infection of *P*. *litchii* stimulate the resistant action by up-regulated *NPR1* and *PP2C*s at early stage of incubation phase in susceptible cultivar.

Interestingly, the profile was reversed, which the four RFOs related genes were up-regulated whereas the *GSO2* and *SDR1* were down-regulated in ‘Guiwei’ at 24 hpi. Similarly, the profile of phenylpropanoid metabolism, ET related genes, and *PP2C*s at 24 hpi was reversed at 48 hpi. The promotion and repression of resistance both exist at the same time and fluctuated at the next time in susceptible cultivar. It speculates that the successful infection of *P*. *litchii* in susceptible cultivar at penetration phase (6 hpi) restrain the resistance by down-regulated RFOs related genes, which in turn restrain the *GSO2*, *SDR1*, ET-related genes, and phenylpropanoid metabolism related genes at 24 hpi (Fig. S10). Meanwhile, *GSO2* sense the infection of *P*. *litchii* and enhance resistance by up-regulated *SDR1*, which in turn induce resistance by up-regulated RFOs related genes, *NPR1*, and *PP2C*s at 24 hpi. At 48 hpi, RFOs related genes and *PP2C*s were down-regulated due to *GSO2* and *SDR1* down-regulated at 24 hpi. The *GSO2*, *SDR1*, ET-related genes, and phenylpropanoid metabolism related genes were up-regulated due to RFOs related genes up-regulated at 48 hpi. This trend suggests that susceptible cultivar experiencing *P*. *litchii* infection fundamentally repression of RFOs at penetration phase and disturbance signal transduction by altered endogenous hormone balance at incubation phase.

The pathogen infected unsuccessfully exocarp of ‘Heiye’ until 6 hpi. At this time, a relative abundance of DEGs interacted with *P*. *litchii* were detected, indicating that response to infection of *P*. *litchii* is strong in resistant cultivar. Chitin is a typical pathogen-derived molecule from fungal cell walls which elicits plant immune responses. The former study indicated that the cell of oomycete species was composed of lignin as the major component and with few chitins or without chitin. As markers of response to fungal infection, five chitinase genes were up-regulated in ‘Heiye’ after inoculation with *P*. *litchii*. Higher up-regulation of chitinase genes might be a consequence of recognition pathogen and subsequent activation of pathogen reaction. In actual, the expression of five chitinases was lower in resistance cultivar than in susceptible cultivar. Therefore, the increasing of chitinases in ‘Heiye’ is not an effective reaction to block infection of *P*. *litchii* but a biomarker effectively linked to defense response of litchi. SAMT converts SA to SA methyl ester (MSA), which act as an airborne signal that triggers defense responses in uninfected plants. *SAMT* was induced specifically around the lesions. In this study, one *SAMT* was down-regulated in ‘Heiye’ at 6 hpi. It suggests that the SA-mediated resistance is not the major mechanism against *P*. *litchii* in resistant cultivar.

Plant employ PRRs (RLKs and RLPs) for sensitive and rapid detection of the potential danger caused by pathogens. RLKs is surface-localized and contain various ligand-binding ectodomains that perceive PAMPs. RLKs contain three functional domains: an extracellular domain, a transmembrane domain, and an intracellular serine/threonine kinase domain. External signal ligands are recognized by the extracellular domain which triggers phosphorylation activity of the intracellular cytoplasmic kinase domain. The intracellular cytoplasmic kinase domain then activates the downstream signaling pathways. One *RLP* and six *RLK*s were up-regulated in resistance cultivar at early infection phase (6 hpi). One *RLK* with a mannose-binding domain was regulated at three time-points, which was up-regulated (FC = 2.28) at 6 hpi. LRR receptor-like serine/threonine-protein kinase (LRR-RLKs) regulates cell wall composition and structure and required for callose deposition upon infection in *Arabidopsis*^37^. LRR-RLKs confers resistance to the pathogenic bacteria *Ralstonia solanacearum* and to the necrotrophic fungi *Plectosphaerella cucumerina*^38–40^. The rice receptor-like kinase protein Xa21 confers resistance to bacterial blight disease, caused by infection of *Xanthomonas oryzae pv*. *oryzae (Xoo)*^41^. Xa21 perceives the presence of *Xoo* and relays the signal to the nucleus through multi-step signal cascades involving some key proteins such as XA21 Binding Protein 3 (XB3), mitogen-activated protein kinase 5 (MAPK5), and transcription factors (TFs) including *OsWRKY62*. XB3 is an E3 ubiquitin ligase protein that binds to the kinase domain of XA21^13^.

Interaction of E3 ligase proteins with the kinase domain of RLKs appears to be a conserved mechanism for the regulation of various plant processes^42^. Protein degradation mediated plays critical roles in plant immunity. The ubiquitin-proteasome system (UPS) is used for selectively degrading proteins, in which E3 ligases determine the substrate specificity^43^. E3 ubiquitin ligases can be classified into different groups based on the presence of specific HECT, RING, or U-box domains (PUBs)^43^. There were seven up-regulated protease genes in ‘Heiye’, which including one E3 ubiquitin ligase, two *PUB*s, two *SBT*s, and one *NEDD8*-specific protease. E3 ubiquitin ligases have been involved in the pathogen perception, as they appear to modulate the PPRs. In addition, E3 ubiquitin ligases are also involved in the signaling conduction. OsBBI1 is an E3 ubiquitin ligase protein that mediate broad-spectrum disease resistance against the blast fungus in rice. *OsBBI1*-overexpressing plants accumulate hydrogen peroxide and phenolic compounds and display enhanced cross-linking of proteins in cell walls at infection sites by *Magnaporthe oryzae*^44^. CaRING is another active E3 ligase in pepper and is induced by an avirulent strain of *Xanthomonas campestris pv vesicatoria*^45^. *CaRING* overexpression in *Arabidopsis* induces enhanced resistance to *Pseudomonas* and *Hyaloperonospora arabidopsidis*^46^. *PUB17* is positive regulators of ETI responses in *Arabidopsis*. Plant over-expressing *OsPUB15* at early stage in rice display a constitutive activation of plant basal defense responses, including excessive accumulation of hydrogen peroxide, up-regulated expression of *PR*^47^. *SBT*s are serine proteases that fulfill highly specific functions in plant development and signaling cascades^48^. It has been shown that several *SBT*s are specifically induced following pathogen infection^48^. *SBT3*.*3* was hypothesized to function as a receptor located in the plasma membrane activating downstream immune signaling processes in *Arabidopsis*^48^. NEDD8-specific protease 1 processes the preform of the ubiquitin-like protein, while their function related to pathogen was not reported^49^.

The proteasome pathway plays a key role in turning off transcription mediate. Transcription factor families commonly reported to be involved in plant defense. Four TFs were up-regulated during pathogen infection in ‘Heiye’. Two TF (*CYCD3-1* and *OFP4*) involved in regulation of cell wall remodeling were up-regulated in ‘Heiye’ at 6 hpi. *CYCD3-1* involved in the control of the cell cycle at the G1/S transition and the induction of mitotic cell division. Plants overexpressing *CYCD3-1* show extensive leaf curling and disorganized meristems^50^. *CYCD3-1* is mediated by a proteasome-dependent pathway. *OFP4* forms a transcription repression complex with KNAT7 to regulate secondary cell wall formation. Plants over-expressing OFP4 show curled leaves in *Arabidopsis*^51^. *CYCD3-1* and *OFP4* may play an important role in regulation of cell wall remodeling to block the infection of pathogen in resistant cultivar at early stage. Moreover, several functional DEGs related to cell wall remodeling respond to infection of *P*. *litchii* in ‘Heiye’ at 6 hpi. Expansin-A4 can loosen and extension of plant cell walls by disrupting non-covalent bonding between cellulose microfibrils and matrix glucans^52^. *CSLA2* possesses biosynthesis galactomannan, which is a non-cellulosic polysaccharides of plant cell wall^53^. Glucan 1,3-beta-glucosidase was involved in plasmodesmal callose degradation and implicated in the defense of plants against pathogens^54^. *F6’H2* was involved in scopoletin biosynthesis (included in Phenylproanoid biosynthesis)^55^. Another gene, *KCS1*, contributes to cuticular wax and suberin biosynthesis^56^. It suggests that the inoculation with *P*. *litchii* lead to cell wall remodeling in resistant cultivar at early stage.

Another up-regulated transcription factor *bHLH100* belongs to a basic helix-loop-helix DNA-binding superfamily. *Arabidopsis* transformants overexpressing *bHLH100* showed increased tolerance to high Zn and nickel compared to wild-type plants, confirming their role in metal homeostasis^57^. The high expression of *bHLH* explain the mechanism of resistance to *Dryocosmus kuriphilus* in *Castanea mollissima* Shuhe-WYL strain^58^. *NaMYC2*, a member of bHLH family, enhance resistance by regulating the biosynthesis of nicotine and phenolamides in *Nicotiana attenuate*^59^. On the other hand, the mutant of *Arabidopsis*, *bhlh99*, exhibited enhanced disease susceptibility to both *Botrytis cinerea* and *Plectosphaerella cucumerina*^60^. It speculates that the up-regulated *bHLH100* will enhance disease resistance against *P*. *litchii* in resistance cultivar.

In generally, ‘Guiwei’ has a feeble response to infection of *P*. *litchii* due to lagging transcriptional modulation at early incubation stage. Once the infection was successful, *P*. *litchii* repressed resistant action in ‘Guiwei’ by down-regulated genes involved in phenylpropanoid metabolism, ET biosynthesis and signaling conduction. Whereas, ‘Heiye’ resist infection through sensing pathogens by receptor-like kinases at early infection stage, upon which they activate defense responses, such as transcription factors, degradation of proteasome, that lead to cell wall remodeling and activating expression of resistance genes to prevent disease.

## Methods

### Pathogen

The pathogen of *P*. *litchii* isolate (hk-1) was preserved in our lab. The isolate was cultured on 10% V8 agar media for 7 days at 25 ± 2 °C. The plates were flooded with 10 mL of sterile water and kept for 2 hour at 4 °C to promote zoospores release. The spore suspension was filtrated through double sterile layer and adjusted to 1 × 10^4^ spores·mL^−1^ for inoculation.

### Plant materials and treatment

In present study, freshly harvested mature fruits of *Litchi chinensis* cv. ‘Guiwei’ and ‘Heiye’ were obtained from orchards in Zhanjiang, China from 2012 to 2017. Fruits were selected for uniformity of shape, color, size and free of blemish or disease. Destalking of fruits was done by sharp scissor leaving about 4 mm pedicel. Prior to inoculation, fruits were precleaned with sterile water for twice. The fruits of each cultivar were divided into two groups, of which infiltrated respectively into a solution contained sterile water (mock-inoculation) or 1 × 10^4^ spores·mL^−1^ spore suspension of *P*. *litchii* (inoculation) for 10 min. After air-drying, the fruits of each group were divided into three biological replications, which were placed in polyethylene packets (280 × 200 × 150 mm, 20 fruits per packet), and stored with 85–90% relative humidity at 25 ± 2 °C. At 2016, the pericarps of thirty fruits inoculated with *P*. *litchii* from each replication were collected respectively at 0, 6, 24, 48, 72, and 96 hpi, as well as mock-treated samples. Samples for SEM study was immediately treated as descripted by Hong *et al*.^61^. Samples for RNA isolated were stored immediately in liquid nitrogen and then stored at −80 °C until use.

### Disease index

The DI was calculated according to the standard described by Cao *et al*.^62^ and with little modification. The disease grade was measured by monitored 60 fruits at 72 hpi. The disease grade was recorded the percentage of pathogen infection and accessed by the following scale: 0 = no disease; 1 = less than 1/10 disease; 3 = 1/10 to 1/4 disease; 5 = 1/4 to 1/2 disease; 7 = 1/2 to 3/4 disease; and 9 = more than 3/4 disease. The disease index was calculated as:

$$\mathrm{DI}=\sum ({\rm{diseasescale}}\times {\rm{percentageofcorrespondingfruitswithineachgrade}})\times {\rm{100}}$$

Disease resistance levels of the different genotypes were classified as: Highly Resistant (HR: scores of 0–25.00); Resistant (R: scores of 25.01–50.00); Susceptible (S: scores of 50.01–75.00); or Highly Susceptible (HS: scores of 75.01–100.00)^62^.

### Scanning electron microscopy

We observe the disease process on the exocarp of two cultivars by SEM. According to methods outlined by Hong^61^, pericarp samples (5 mm × 5 mm) were fixed in 4% glutaraldehyde at 4 °C for over 6 hour, and then washed three times with 0.1 M phosphate buffer solution (PBS) for 10 min. The samples were then transferred into 1% osmic acid at 4 °C for 2 hour and washed three times with 0.1 M PBS for 10 min. The samples were then dehydrated with a series of ethanol mixtures (30%, 50%, 70%, 85%, and 100%) for 20 min respectively, followed by a series of tert‐butyl alcohol mixtures (50%, 75%, 100%) twice to remove the ethanol. After being dried, samples were sprayed with a 12.5–15 nm gold layer. Samples were examined and photographed using a HITACHI S-4700 Scanning Electron Microscope at an accelerating voltage of 2.0 kV and working distance of 12.0 mm.

### RNA Extraction and Sequencing

Total RNA was extracted from 6, 24, and 48 hpi sample of three biological replicates using the Quick RNA Isolation Kit (Huayueyang, China) according to the manufacturer’s instructions. DNAase I (Takara, Otsu, Japan) was given to remove genomic DNA contamination. The integrity of total RNA was verified through RNase-free agarose gel electrophoresis, and the concentration was measured using a 2100 Bioanalyzer (Agilent Technologies, Santa Clara, CA, USA). A cDNA library was constructed for high-quality RNA (2 μg) from each sample and was sequenced with Illumina HiSeq™ 2500 (San Diego, CA, USA) using a 125 paired-end module at Berry Genomics Corporation (Beijing, China, http://www.berrygenomics.com).

### *De novo* assembly

Before assembly, adapter sequences were removed from the raw reads. Low-quality reads (>50% bases with quality scores ≤5) and unknown bases (>10% N bases) were removed from each dataset. The high-quality clean reads from all 36 samples is too huge to assemble. To reduce memory requirements and improve upon runtimes, three runs of in silico normalization were carried out prior to assemble. The sequences from each cultivar were respectively normalized using insilico_read_normalization.pl in Trinity with default parameters, and then the former results were normalized finally^63^. The reduced reads were *de novo* assembled using Trinity pipeline (Trinity-v2.4.0)^63^. The contigs were further processed with cd-hit for removes redundancy^64^. The normalization reads were mapping to the de-redundancy contigs with botiwe2 and calculated the expression with RSEM^65,66^. The isoforms with highest expression were selected as candidate genes and merged via corset to construct unique consensus sequences (unigenes) as ref.^67^.

### Function annotation and DEG analysis

The assembled unigenes were annotated by BLASTx (E-value < 10^−5^) against the NCBI non-redundant (Nr) database^68^, the Swiss-Prot protein database (Swiss-Prot)^69^, GO^19^, and KEGG^20^. The sequencing reads for each sample were remapped to the reference sequences using bowtie2^65^. After the number of reads mapped to each unigene was counted, differential expression analysis was conducted using edgeR^70^. The library was recalibrated, and the lowly expressed genes (<5 counts) were filtered in each sample. The threshold of DEGs was set as |log2FC| ≥ 1, P-value < 0.001, and FDR < 0.01 in each comparison.

Plants of two litchi cultivars ‘Guiwei’ (susceptible; referred as ‘G’) and ‘Heiye’ (resistant; referred as ‘H’) were either inoculated (‘I’) with the pathogen (GI and HI), or mock-inoculated (‘C’) with sterile deionized water (GC and HC) at three time-points (6, 24, and 48 hpi). For example, the ‘GC_6 hpi’ represent the sample of ‘Guiwei’ with mock-inoculated at 6 hpi. To infer the transcriptional changes in the two genotypes, DEGs were respectively identified by comparing the expression levels bewteen ‘Heiye’ and ‘Guiwei’ with mock-treated (HC_vs_GC) at 6, 24, and 48 hpi. For convenience, DEGs showing higher expression levels in ‘Heiye’ were designated ‘up-regulated’, whereas the rest were designated ‘down-regulated’. To infer the transcriptional changes responding to infection of *P*. *litchii*, DEGs were respectively identified by comparing the expression levels between inoculation and mock-inoculation in cultivar (HI_vs_HC or GI_vs_GC) at 6, 24, and 48 hpi. DEGs showing higher expression levels in inoculation with *P*. *litchii* were designated ‘up-regulated’, whereas the rest were designated ‘down-regulated’. The DEGs of each comparison was further subjected to GO and KEGG enrichment analysis to verify biological significance with the threshold (Q-value ≤ 0.05). PCA was performed on the normalized gene expression values using R statistical software (version 3.1.0)^71^. The heat map was constructed using the R package ‘pheatmap’^72^.

### Real time PCR validation

Total RNA was extracted as above description. Two μg of RNA was reverse-transcribed using M-MLV reverse transcriptase (Invitrogen, USA) and an oligo (dT18) primer according to the manufacturer’s protocol. Six DEGs involved in ‘Phenylpropanoid biosynthesis’ were analysed using qRT-PCR. Primers for selected transcripts were designed by Primer3 (http://frodo.wi.mit.edu/primer3)(Table S12). The qRT-PCR reactions were performed with 10 μL of SYBR Green master mix, 50 ng of cDNA, and 500 nM each of the sense and antisense primers, in a total volume of 20 μL (Takara). Actin was used as an internal control for calculating relative abundances using the 2^−△△CT^ method. The expression ratio was the average of three technical replicates and three biological replicates.

## Supplementary information

Supplementary materials

## Data Availability

Additional data sets generated and/or analysed during the current are available from the corresponding author on reasonable request.
