# Supplementary material for: Early responses given distinct tactics to infection of Peronophythora litchii in susceptible and resistant litchi cultivar
Source: Sci Rep. 2019 Feb 26;9:2810. doi: 10.1038/s41598-019-39100-w (PMC6391439; doi:10.1038/s41598-019-39100-w)
Supplement: Supplementary file 1 — Supplementary materials [file 41598_2019_39100_MOESM1_ESM.pdf]

Early responses given distinct tactics to infection of *Peronophythora litchii* in susceptible and resistant litchi cultivar

Jinhua Sun[1], Lulu Cao[1], Huanling Li[1], Guo Wang[1], Shujun Wang[1], Fang Li[1], Xiaoxiao Zou[2], and Jiabao Wang[1*]

[1]The Environment and Plant Protection Institute, Chinese Academy of Tropical Agricultural Sciences, Haikou, PR China

[2]The Institute of Tropical Bioscience and Biotechnology, Chinese Academy of Tropical Agricultural Sciences, Haikou, PR China

[*]Correspondence to fdabo@163.com

Supplementary material

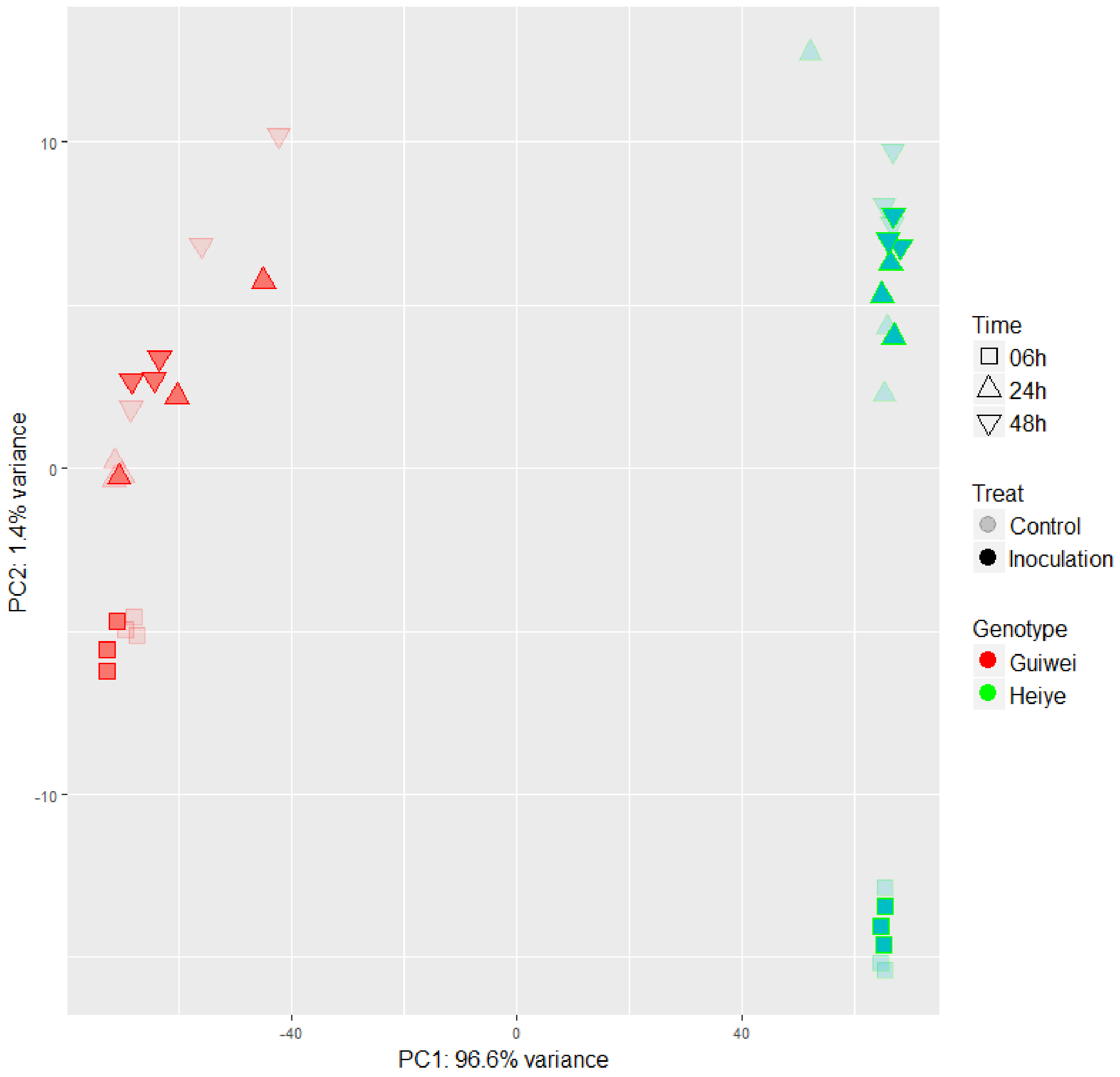

Figure S1 Principal component analysis of litchi transcriptome data. The expression values for 32614 genes in twelve types (two cultivars, two treatments, and three time-points) are projected onto the two principal components. The first principal component separates resistant cultivar ‘Heiye’ from susceptible cultivar ‘Guiwei’, while the second principal components show a further separation between 6 hpi and the others in ‘Guiwei’.

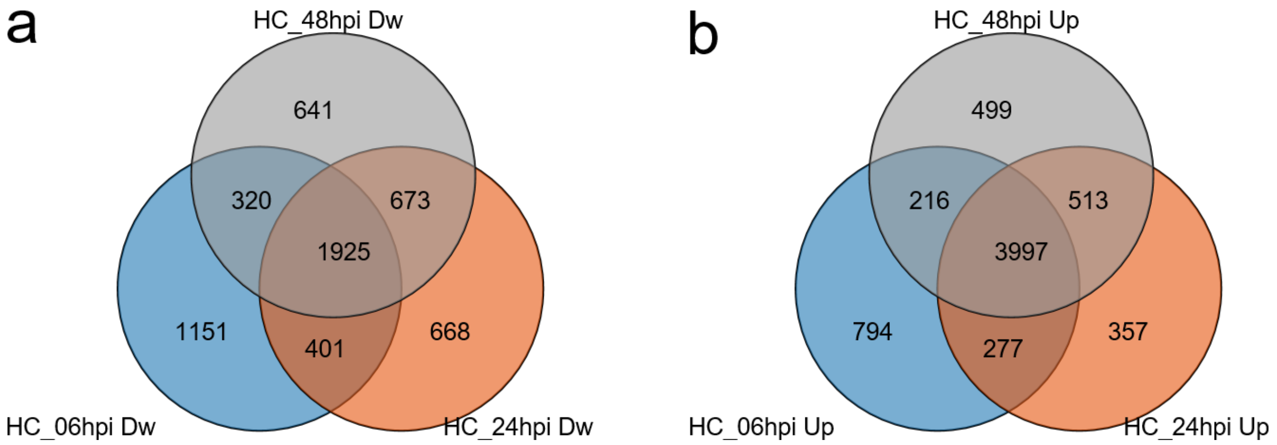

Figure S2 Down- and up-regulated DEGs among three time-points in two cultivars. a. the down-regulated DEGs among three time-points in two cultivars with mocked-inoculation. b. the up-regulated DEGs among three time-points in two cultivars with mocked-inoculation.

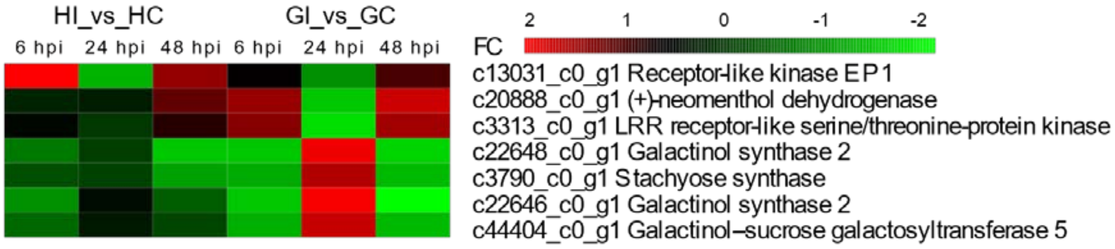

Figure S3 The DEGs respond to *P. litchii* in 'Guiwei' and 'Heiye' at three time-points.

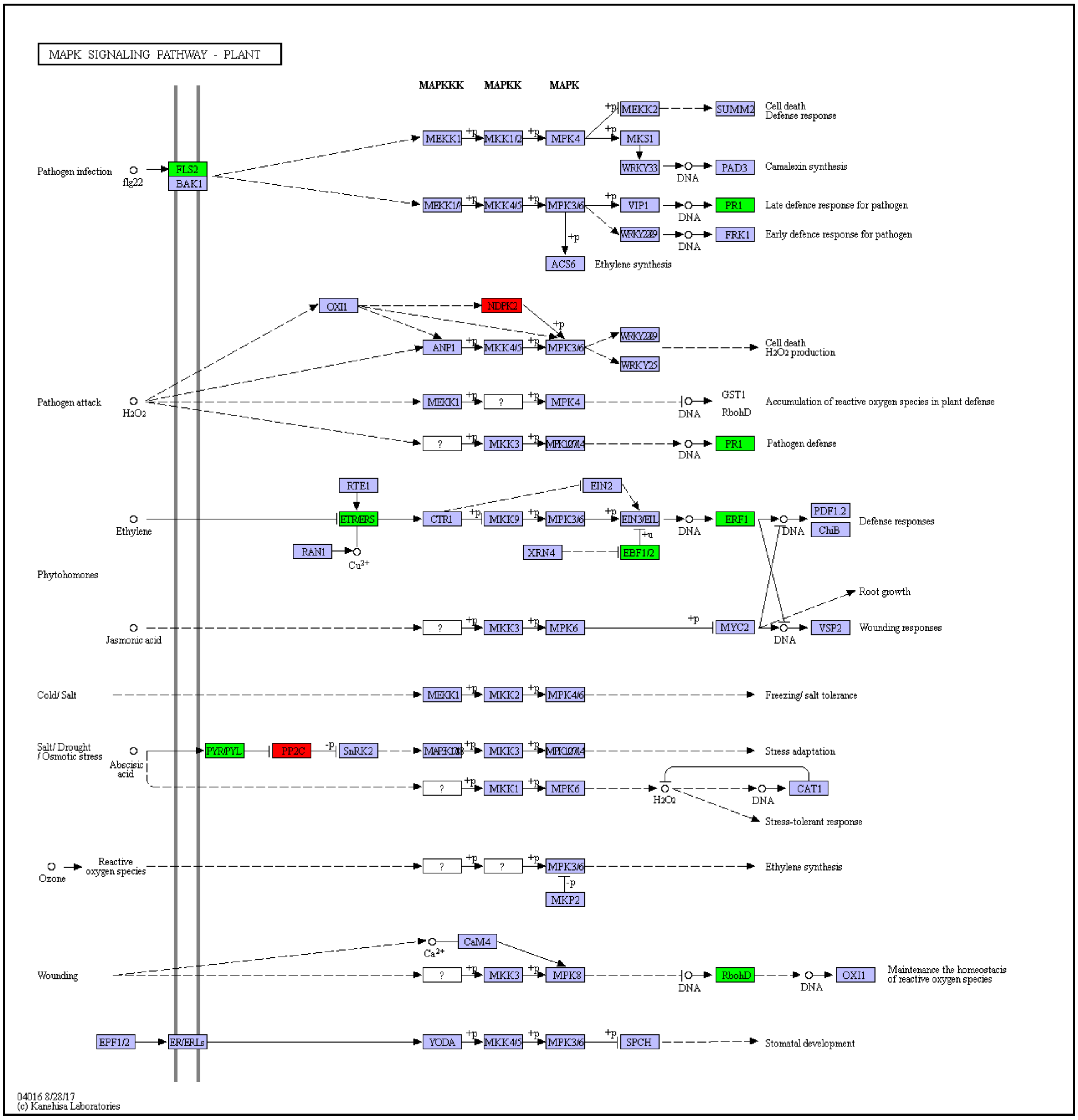

Figure S4 'MAPK signaling pathway – plant' was suppressed under *P. litchii* infection in 'Guiwei' at 24 hpi. KEGG pathway map (ko04016) is adapted here from http://www.kegg.jp/kegg/kegg1.html. The KEGG database has been described previously[20].

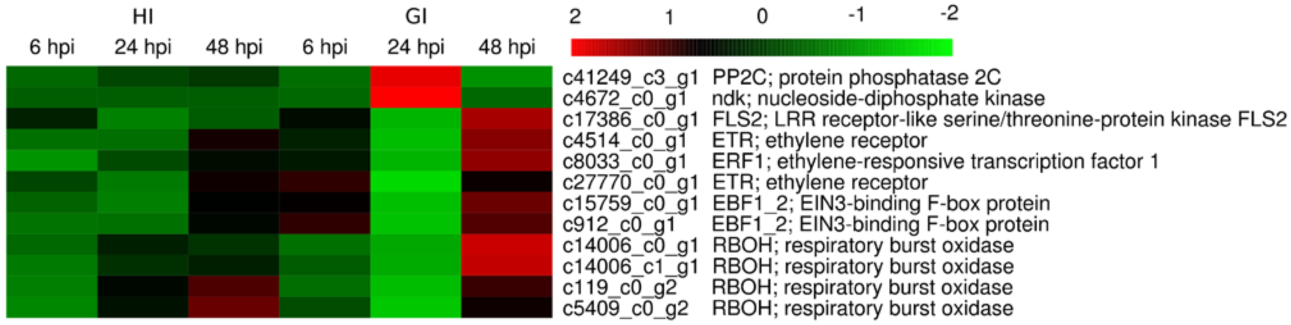

Figure S5 Cluster of DEGs involved in 'MAPK signaling pathway – plant' in 'Guiwei' at 24 hpi.

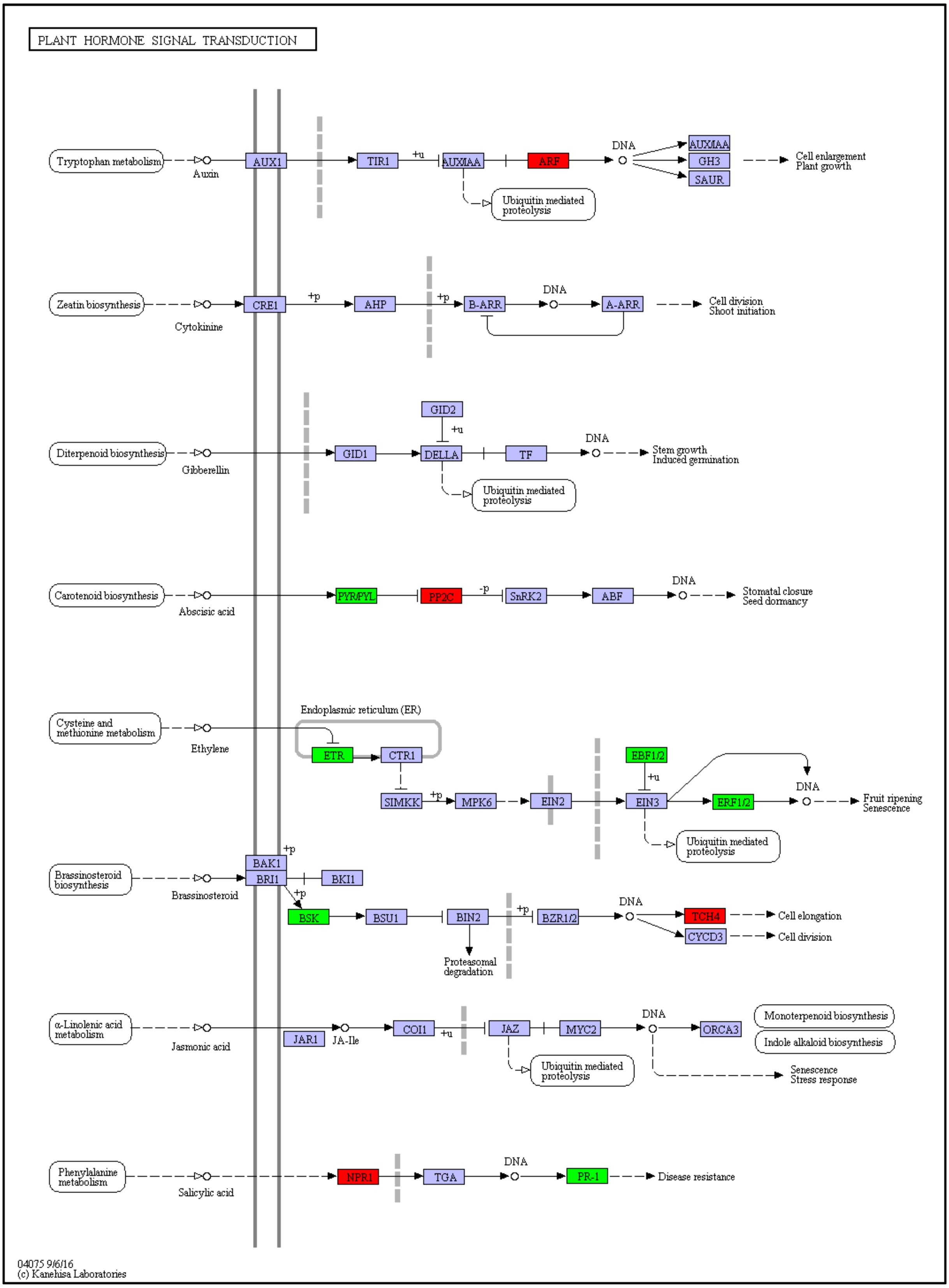

Figure S6 'Plant hormone signal transduction' was suppressed under *P. litchii* infection in 'Guiwei' at 24 hpi. KEGG pathway map (ko04075) is adapted here from http://www.kegg.jp/kegg/kegg1.html. The KEGG database has been described previously[20].

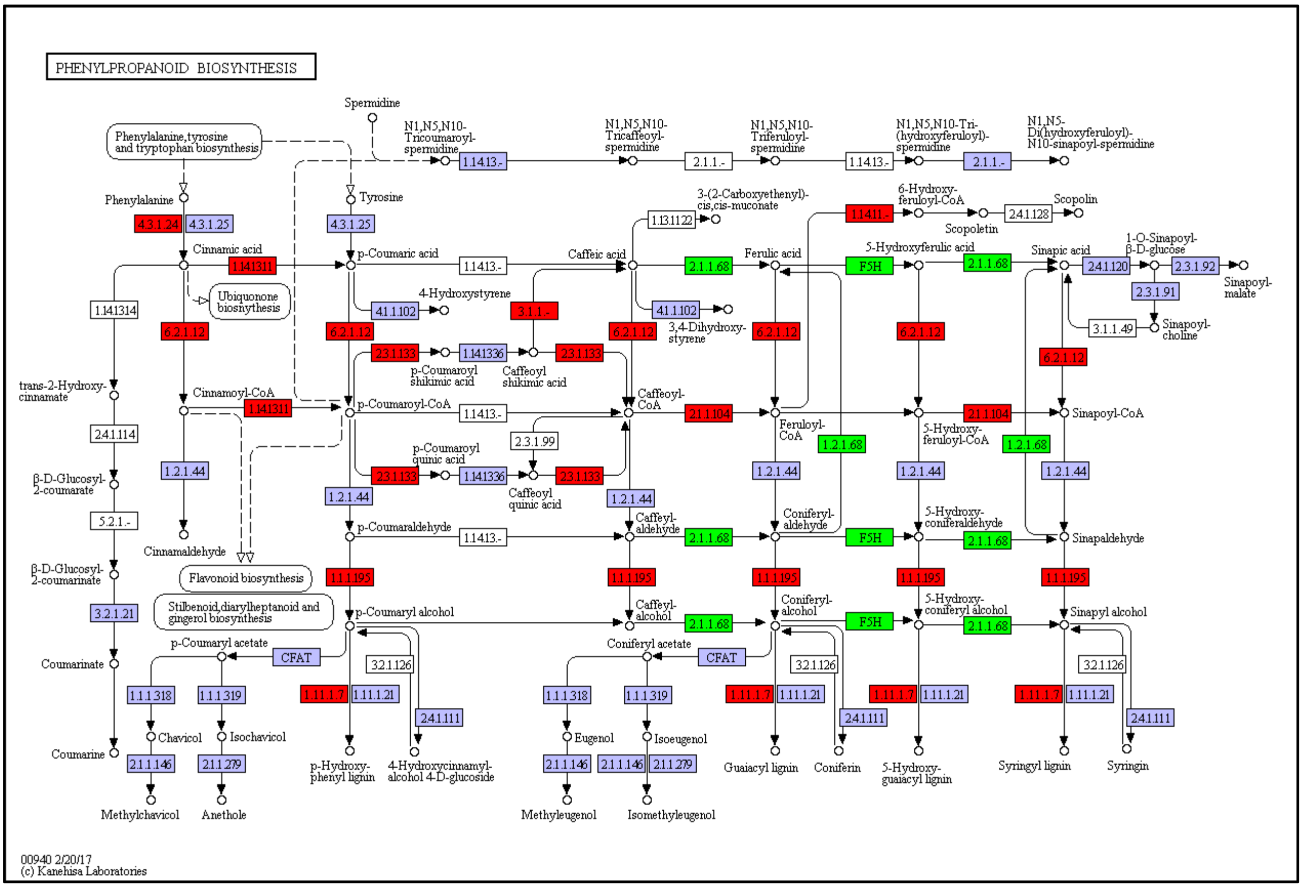

Figure S7 'Phenylpropanoid biosynthesis' was suppressed under *P. litchii* infection in 'Guiwei' at 24 hpi. KEGG pathway map (ko00940) is adapted here from http://www.kegg.jp/kegg/kegg1.html. The KEGG database has been described previously[20].

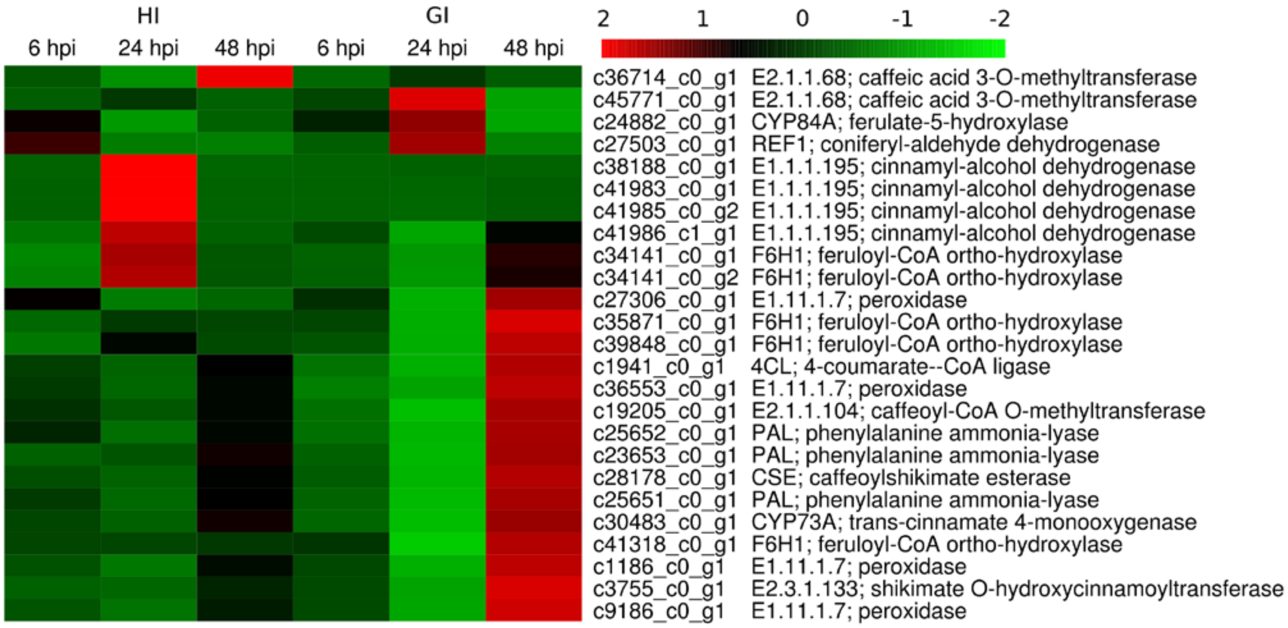

Figure S8 Cluster of DEGs involved in 'Phenylpropanoid biosynthesis' in 'Guiwei' at 24 hpi.

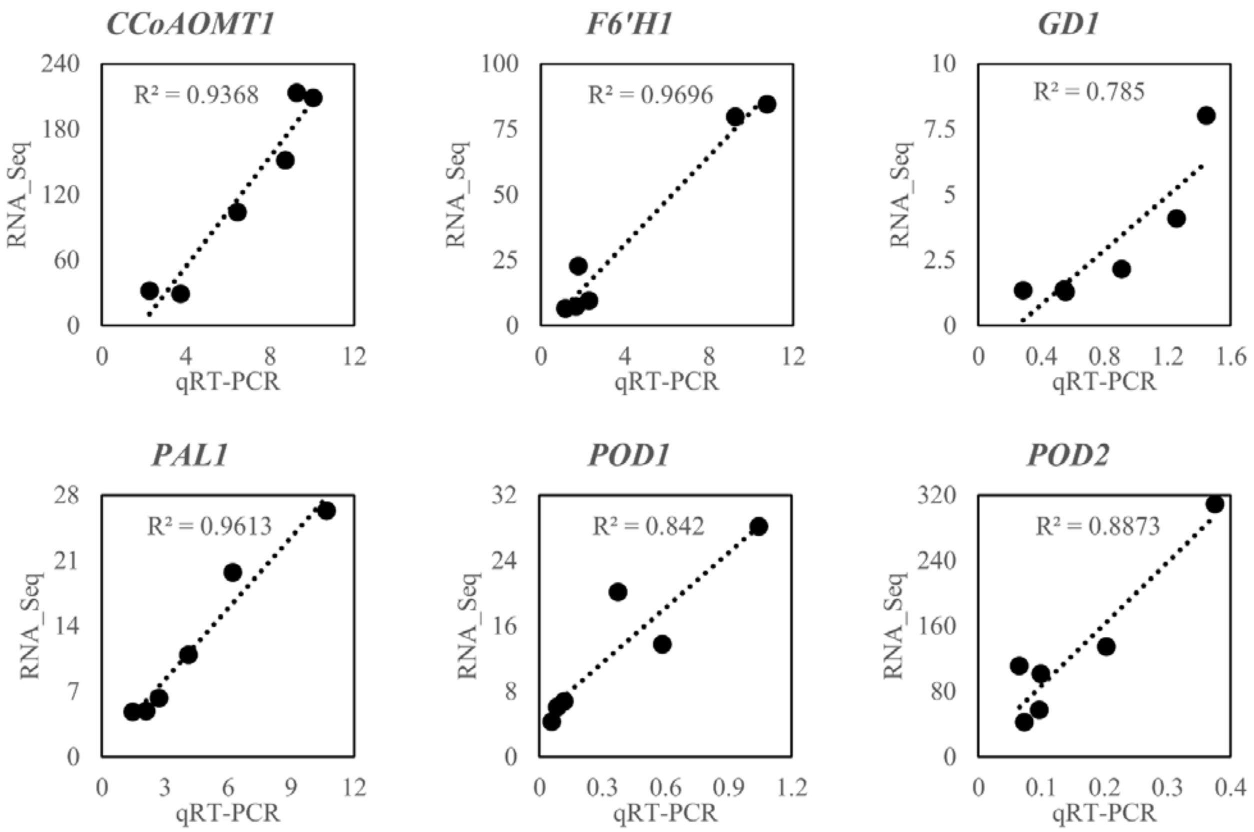

Figure S9 Validation of gene expression of six selected genes by qRT-PCR. *CCoAOMT1*, Caffeoyl-CoA O-methyltransferase 1; *F6'H1*, Feruloyl-CoA ortho-hydroxylase 1; *GD1*, Beta-glucosidase 1; *PAL1*, Phenylalanine ammonia-lyase 1; *POD1*, Peroxidase 1; *POD2*, Peroxidase 2. Scatter plots shows the correlation between PFMK by RNA-Seq and relative expression by qRT-PCR.

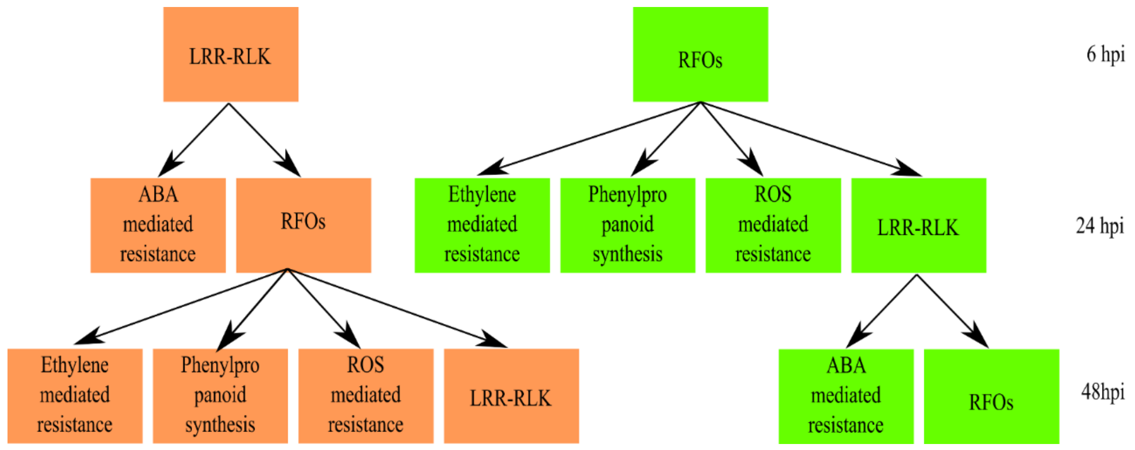

Figure S10 The demonstration of response to infection of *P. litchii* in susceptible cultivar.

Table S1 Disease evaluation of 'Guiwei' and 'Heiye' responses to *P. litchii* in six years.

Cultivars	2012		2013		2014		2015		2016		2017	
	DI [a]	DRL [b]	DI [a]	DRL [b]	DI [a]	DRL [b]	DI [a]	DRL [b]	DI [a]	DRL [b]	DI [a]	DRL [b]
Guiwei	55.2	S	58.2	S	51.4	S	55.7	S	53.0	S	64.81	S
Heiye	26.5	R	32.6	R	20.3	R	33.0	R	22.8	R	15.79	R

[a] Disease Index

[b] Disease Resistance Levels

Table S2 Sequencing statistics for the thirty-six samples of two cultivars at three time-points.

Cultivars	Samples ID	Experimental condition	Sample time (hpi)	Clean Data (G)	No. of clean reads pairs (10^6)	No. of mapped reads pairs (10^6)	Mapped percentage (%)
Guiwei	GC_6hpi_1	Mock-inoculation	6	3.48	13.91	9.53	68.54
	GC_6hpi_2	Mock-inoculation	6	3.43	13.74	9.3	67.67
	GC_6hpi_3	Mock-inoculation	6	3.91	15.62	10.71	68.56
	GC_24hpi_1	Mock-inoculation	24	3.69	14.75	9.9	67.17
	GC_24hpi_2	Mock-inoculation	24	3.88	15.53	10.19	65.63
	GC_24hpi_3	Mock-inoculation	24	3.38	13.53	8.97	66.34
	GC_48hpi_1	Mock-inoculation	48	4.16	16.62	11.46	68.93
	GC_48hpi_2	Mock-inoculation	48	4.03	16.12	11.09	68.76
	GC_48hpi_3	Mock-inoculation	48	3.49	13.95	9.86	70.67
	GI_6hpi_1	Inoculation	6	4.09	16.38	11.35	69.32
	GI_6hpi_2	Inoculation	6	3.71	14.83	9.98	67.29
	GI_6hpi_3	Inoculation	6	4.22	16.89	11.47	67.88
	GI_24hpi_1	Inoculation	24	3.85	15.4	10.11	65.67
	GI_24hpi_2	Inoculation	24	3.27	13.09	8.57	65.42
	GI_24hpi_3	Inoculation	24	2.98	11.93	7.98	66.87
	GI_48hpi_1	Inoculation	48	3.71	14.84	10.34	69.73
	GI_48hpi_2	Inoculation	48	3.49	13.95	9.62	69.01
	GI_48hpi_3	Inoculation	48	3.67	14.69	10.23	69.61
Heiye	HC_6hpi_1	Mock-inoculation	6	3.64	14.56	10.23	70.28
	HC_6hpi_2	Mock-inoculation	6	3.63	14.53	10.48	72.13
	HC_6hpi_3	Mock-inoculation	6	3.58	14.34	10.35	72.17
	HC_24hpi_1	Mock-inoculation	24	3.65	14.61	9.59	65.6
	HC_24hpi_2	Mock-inoculation	24	3.56	14.22	9.33	65.57
	HC_24hpi_3	Mock-inoculation	24	3.23	12.93	8.1	62.64
	HC_48hpi_1	Mock-inoculation	48	3.85	15.39	10.09	65.53
	HC_48hpi_2	Mock-inoculation	48	3.7	14.78	9.61	65.04
	HC_48hpi_3	Mock-inoculation	48	3.32	13.28	8.64	65.08
	HI_6hpi_1	Inoculation	6	4.34	17.35	12.38	71.37
	HI_6hpi_2	Inoculation	6	4.15	16.61	12.08	72.71
	HI_6hpi_3	Inoculation	6	3.5	14.01	9.51	67.88
	HI_24hpi_1	Inoculation	24	3.61	14.46	9.63	66.6
	HI_24hpi_2	Inoculation	24	4.08	16.32	10.44	63.99
	HI_24hpi_3	Inoculation	24	3.16	12.63	8.26	65.42
	HI_48hpi_1	Inoculation	48	4.08	16.33	10.73	65.69
	HI_48hpi_2	Inoculation	48	3.24	12.96	8.5	65.55
	HI_48hpi_3	Inoculation	48	4.11	16.45	11.44	69.59

Table S3 Overview of functional annotation of the litchi transcriptome.

Database	Number of annotated genes	Percentage (annotated/total number of genes, %)
Nr Annotation	29963	91.87
String Annotation	28231	86.56
SwissProt Annotation	23515	72.10
PFAM Annotation	20404	62.56
GO Annotation	22588	69.26
KEGG Annotation	6593	20.22
Total annotated genes	30006	92.00

Table S4 Significantly enriched GO term of 5922 common DEGs between two cultivars at three time-points.

GO_term	GO_ID	Down_regulated	Up_regulated	All_DEGs	Background genes
Oxidation-reduction process	GO:0055114	135	317*	452*	2174
Defense response	GO:0006952	91*	79	170*	691
Aminoglycan metabolic process	GO:0006022	15*	11	26*	63
Glucosamine-containing compound catabolic process	GO:1901072	14*	10	24*	58
Glucosamine-containing compound metabolic process	GO:1901071	14*	10	24*	58
Amino sugar catabolic process	GO:0046348	14*	10	24*	58
Aminoglycan catabolic process	GO:0006026	14*	10	24*	58
Chitin metabolic process	GO:0006030	14*	10	24*	58
Chitin catabolic process	GO:0006032	14*	10	24*	58
Secondary metabolic process	GO:0019748	26	49	75*	278
Non-recombinational repair	GO:0000726	6	10	16*	32
Double-strand break repair via nonhomologous end joining	GO:0006303	6	10	16*	32
Amino sugar metabolic process	GO:0006040	15*	13	28*	75
Secondary metabolite biosynthetic process	GO:0044550	19	36	55*	192
Response to stress	GO:0006950	143*	192	335*	1642

* Represent significent enrichment at 0.01 level

Table S5 Significantly enriched KEGG pathways of 5922 common DEGs between two cultivars at three time-points.

Kegg ID	No. of down-regulated genes	No. of up-regulated genes	P-value (BH corrected)	Pathway	All	Pvalue	enrich_factor
ko04626	60	80	5.34E-06	Plant-pathogen interaction	140	1.00E-07	1.50
ko00592	4	20	2.33E-02	alpha-Linolenic acid metabolism	24	7.27E-04	1.92
ko00270	14	25	2.08E-02	Cysteine and methionine metabolism	39	7.80E-04	1.64
ko00905	5	4	2.04E-02	Brassinosteroid biosynthesis	9	8.91E-04	3.05
ko00901	0	4	1.81E-02	Indole alkaloid biosynthesis	4	9.06E-04	5.76
ko00942	2	6	2.91E-02	Anthocyanin biosynthesis	8	1.64E-03	3.07
ko00350	10	10	4.87E-02	Tyrosine metabolism	20	3.05E-03	1.86

Table S6 Common DEGs involved in 'Plant-pathogen interaction' between 'Heiye' and 'Guiwei'.

Ko ID	No. of down-regulated genes	No. of up-regulated genes	Gene_name	Pathway
K01373	0	4	CTSF	cathepsin F
K02183	0	2	CALM	calmodulin
K05391	8	10	CNGC	cyclic nucleotide gated channel, plant
K09422	8	7	MYB	transcription factor MYB, plant
K13412	1	1	CPK	calcium-dependent protein kinase
K13414	1	0	MEKK1	mitogen-activated protein kinase kinase kinase 1
K13416	0	1	BAK1	brassinosteroid insensitive 1-associated receptor kinase 1
K13420	0	1	FLS2	LRR receptor-like serine/threonine-protein kinase
K13425	0	1	WRKY22	WRKY transcription factor 22
K13427	1	0	NOA1	nitric-oxide synthase, plant
K13429	0	3	CERK1	chitin elicitor receptor kinase 1
K13448	1	6	CML	calcium-binding protein
K13449	0	1	PR1	pathogenesis-related protein 1
K13456	0	1	RIN4	RPM1-interacting protein 4
K13457	6	2	RPM1	disease resistance protein
K13459	30	38	RPS2	disease resistance protein
K15397	3	1	KCS	3-ketoacyl-CoA synthase
K18875	0	1	EDS1	enhanced disease susceptibility 1 protein
K18880	1	0	HCD1	very-long-chain (3R)-3-hydroxyacyl-CoA dehydratase

Table S7 Common DEGs respond to *P. litchii* in cultivars at 24 hpi.

Time (hpi)	Gene name	Fold change of HI_vs_HC (log_2)	Fold change of HI_vs_GI (log_2)	Annotation
24	c36949_c0_g1	1.48	1.33	Protein EXORDIUM-like 2
	c8306_c0_g2	1.67	1.44	Protein EXORDIUM-like 2
	c36893_c0_g1	2.21	1.82	Allene oxide cyclase
	c13847_c0_g1	1.35	1.31	Unkown
	c5397_c0_g3	-2.05	-2.12	Unkown

Table S8 Significantly enriched KEGG pathways of DEGs respond to *P. litchii* infection in litchi.

Comparison	Kegg ID	No. of down-regulated genes	No. of up-regulated genes	P-value (BH corrected)	Pathway	All	Pvalue	enrich_factor
GI_vs_GC_06hpi	ko00052	4	0	3.10E-06	Galactose metabolism	96	6.19E-07	45.78
	ko00902	0	2	3.25E-04	Monoterpenoid biosynthesis	20	1.30E-04	109.88
GI_vs_GC_24hpi	ko04016	15	6	1.86E-07	MAPK signaling pathway - plant	143	1.83E-09	4.77
	ko00710	2	10	1.90E-04	Carbon fixation in photosynthetic organisms	83	7.44E-06	4.70
	ko00940	17	2	1.46E-03	Phenylpropanoid biosynthesis	232	8.61E-05	2.66
	ko00195	0	8	3.16E-03	Photosynthesis	54	2.17E-04	4.81
	ko00450	6	0	3.23E-03	Selenocompound metabolism	30	2.53E-04	6.50
	ko00902	1	4	3.17E-03	Monoterpenoid biosynthesis	20	2.80E-04	8.12
	ko00270	12	1	2.88E-03	Cysteine and methionine metabolism	137	2.82E-04	3.08
	ko04075	11	8	3.56E-03	Plant hormone signal transduction	262	4.19E-04	2.36
	ko00908	6	0	1.80E-02	Zeatin biosynthesis	46	2.65E-03	4.24
	ko00904	1	3	1.83E-02	Diterpenoid biosynthesis	20	2.87E-03	6.50
	ko01200	6	15	1.74E-02	Carbon metabolism	357	3.08E-03	1.91
	ko00073	0	4	2.62E-02	Cutin suberine and wax biosynthesis	23	4.88E-03	5.65
	ko00531	4	0	2.91E-02	Glycosaminoglycan degradation	24	5.71E-03	5.41
	ko00592	5	2	3.11E-02	alpha-Linolenic acid metabolism	72	6.40E-03	3.16
	ko00052	1	7	4.26E-02	Galactose metabolism	96	9.18E-03	2.71
	ko00360	6	0	4.41E-02	Phenylalanine metabolism	60	9.94E-03	3.25
GI_vs_GC_48hpi	ko00195	15	0	7.28E-08	Photosynthesis	54	2.49E-09	6.71
	ko00940	6	25	1.13E-07	Phenylpropanoid biosynthesis	232	4.84E-09	3.23
	ko00196	9	0	6.89E-07	Photosynthesis - antenna proteins	20	3.53E-08	10.87
	ko04016	11	10	5.67E-06	MAPK signaling pathway - plant	143	3.39E-07	3.55
	ko00710	14	1	1.54E-05	Carbon fixation in photosynthetic organisms	83	1.18E-06	4.36
	ko00902	6	1	1.10E-04	Monoterpenoid biosynthesis	20	9.42E-06	8.45
	ko00360	2	9	3.00E-04	Phenylalanine metabolism	60	2.82E-05	4.43
	ko04075	20	5	7.19E-04	Plant hormone signal transduction	262	7.37E-05	2.30
	ko00904	6	0	1.02E-03	Diterpenoid biosynthesis	20	1.13E-04	7.25
	ko00945	0	5	3.52E-03	Stilbenoid diarylheptanoid and gingerol biosynthesis	17	4.82E-04	7.10
	ko00908	0	8	3.55E-03	Zeatin biosynthesis	46	5.16E-04	4.20
	ko00450	0	6	7.95E-03	Selenocompound metabolism	30	1.22E-03	4.83
	ko00966	3	1	2.75E-02	Glucosinolate biosynthesis	17	4.46E-03	5.68
	ko00910	0	6	4.17E-02	Nitrogen metabolism	42	7.13E-03	3.45
	ko04626	19	15	4.01E-02	Plant-pathogen interaction	534	7.20E-03	1.54
HI_vs_HC_06hpi	ko00520	0	5	3.39E-04	Amino sugar and nucleotide sugar metabolism	196	1.47E-05	14.02
HI_vs_HC_24hpi	ko00520	8	0	4.78E-05	Amino sugar and nucleotide sugar metabolism	196	1.33E-06	9.28
HI_vs_HC_48hpi	ko00196	8	0	1.21E-10	Photosynthesis - antenna proteins	20	2.52E-12	45.47
	ko00904	4	0	3.76E-04	Diterpenoid biosynthesis	20	2.35E-05	22.73
	ko00450	0	4	1.50E-03	Selenocompound metabolism	30	1.25E-04	15.16
	ko00902	1	2	5.30E-03	Monoterpenoid biosynthesis	20	6.62E-04	17.05
	ko00195	4	0	8.43E-03	Photosynthesis	54	1.23E-03	8.42

Table S9 The DEGs involved in ‘MAPK signaling pathway - plant’ respond to attack of *P. litchii* in ‘Guiwei’.

Ko ID	No. of DEGs (Down/Up) at 24 hpi	No. of DEGs (Down/Up) at 48 hpi	Gene_name	Pathway
K20726	0/0	0/1	TMM222	transmembrane protein 222
K13422	0/0	1/0	MYC2	transcription factor
K00940	0/1	1/0	NDK	nucleoside-diphosphate kinase
K14497	0/5	9/0	PP2C	protein phosphatase 2C
K13449	1/0	0/0	PR1	pathogenesis-related protein 1
K14496	1/0	0/0	PYL	abscisic acid receptor PYR/PYL family
K13447	2/0	0/2	RBOH	respiratory burst oxidase
K13420	3/0	0/2	FLS2	LRR receptor-like serine/threonine-protein kinase
K14516	2/0	0/2	ERF1	ethylene-responsive transcription factor 1
K14509	3/0	0/1	ETR	ethylene receptor
K14515	3/0	0/2	EBF1_2	EIN3-binding F-box protein

Table S10 The DEGs involved in 'Plant hormone signal transduction' respond to attack of *P. litchii* in 'Guiwei'.

Ko ID	No. of DEGs (Down/Up) at 24 hpi	No. of DEGs (Down/Up) at 48 hpi	Gene_name	Pathway
K14492	0/0	1/0	ARR-A	two-component response regulator ARR-A family
K14484	0/0	4/0	IAA	auxin-responsive protein
K13422	0/0	1/0	MYC2	transcription factor
K12126	0/0	2/0	PIF3	phytochrome-interacting factor 3
K14488	0/0	1/0	SAUR	SAUR family protein
K14431	0/0	1/0	TGA	transcription factor
K14486	0/1	0/0	ARF	auxin response factor
K14508	0/1	0/0	NPR1	regulatory protein
K14504	0/1	1/0	TCH4	xyloglucan:xyloglucosyl transferase
K14497	0/5	9/0	PP2C	protein phosphatase 2C
K14500	1/0	0/0	BSK	BR-signaling kinase
K13449	1/0	0/0	PR1	pathogenesis-related protein 1
K14496	1/0	0/0	PYL	abscisic acid receptor PYR/PYL family
K14516	2/0	0/2	ERF1	ethylene-responsive transcription factor 1
K14509	3/0	0/1	ETR	ethylene receptor
K14515	3/0	0/2	EBF1_2	EIN3-binding F-box protein

Table S11 The DEGs involved in 'Phenylpropanoid biosynthesis' respond to attack of *P. litchii* in 'Guiwei'.

Ko ID	No. of DEGs (Down/Up) at 24 hpi	No. of DEGs (Down/Up) at 48 hpi	Gene_name	Pathway
K09753	0/0	1/0	CCR	cinnamoyl-CoA reductase
K09755	0/0	1/2	CYP84A	ferulate-5-hydroxylase
K09754	0/0	0/1	CYP98A	coumaroylquinate(coumaroylshikimate) 3'-monooxygenase
K01188	0/0	0/1	GD	beta-glucosidase
K12356	0/0	0/1	UGT72E	coniferyl-alcohol glucosyltransferase
K13066	0/1	1/1	COMT	caffeic acid 3-O-methyltransferase
K01904	1/0	0/2	4CL	4-coumarate--CoA ligase
K00588	1/0	0/1	CCoAOMT	caffeoyl-CoA O-methyltransferase
K18368	1/0	0/1	CSE	caffeoylshikimate esterase
K00487	1/0	0/2	CYP73A	trans-cinnamate 4-monooxygenase
K06892	1/0	0/0	F6'H1	feruloyl-CoA ortho-hydroxylase
K05350	1/0	2/0	GD	beta-glucosidase
K13065	1/0	0/1	HST	shikimate O-hydroxycinnamoyltransferase
K10775	3/0	0/4	PAL	phenylalanine ammonia-lyase
K00430	7/1	1/8	POD	peroxidase

Table S12 Real-time PCR primers for quantification of gene expression.

Unigene_id	Gene_name	Sense_primer	Antisense_primer
NA	Actin	CAACTGGTATTGTCTTGGATTCTG	TCATCAAGGCATCGGTTAGA
c13750	POD2	GCTCAGTTCTTTGAAGCTTTTG	CACTTTGGAGCTGAATATCTCTG
c20636	POD1	AGGGGAGATTAGGCACAGG	ATCATAAACATCACATCCTCACAA
c22119	GD1	ACAATTGAAGAAGGCTATTGATG	ACACAAACTTTTACTGCTCTCTCTC
c26397	CCoAOMT1	CACCTATCCATTGTAACTCCTCTA	TTGGGAAGGTTTTGGAAGAC
c38906	PAL1	TATTGATCCTCTGCTTGATTGTC	GGTTTCCACAAAGTTGACACG
c41318	F6'H1	GCCAGGATCAATTCTTTACC	TACGAATAGTTGCTCTAATTATGAG
